# Swedish national guidelines for diagnosis and management of acute appendicitis in adults and children

**DOI:** 10.1093/bjsopen/zrae165

**Published:** 2025-04-09

**Authors:** Martin Salö, Catarina Tiselius, Anders Rosemar, Elin Öst, Sara Sohlberg, Roland E Andersson

**Affiliations:** Department of Clinical Sciences, Pediatrics, Lund University, Lund, Sweden; Department of Pediatric Surgery, Skåne University Hospital, Lund, Sweden; Department of Surgery, Västmanland Hospital Västerås, Västerås, Sweden; Centre for Clinical Research, Uppsala University, Västerås, Sweden; Department of Surgery, Region Västra Götaland, Sahlgrenska University Hospital Östra, Gothenburg, Sweden; Department of Surgery, Institute of Clinical Sciences, Sahlgrenska Academy, University of Gothenburg, Gothenburg, Sweden; Department of Pediatric Surgery, Karolinska University Hospital, Stockholm, Sweden; Department of Women's and Children's Health, Karolinska Institutet, Stockholm, Sweden; Department of Women´s and Children´s Health, Uppsala University, Uppsala, Sweden; Department of Biomedical and Clinical Sciences, Linköping University, Linköping, Sweden; Futurum Academy for Health and Care, Jönköping County Council, Jönköping, Sweden

## Abstract

**Background:**

Acute appendicitis is one of the most common causes of acute abdominal pain. Differences in the management of this large group of patients has important consequences for the patients and the healthcare system. Controversies regarding the understanding of the natural course of the disease, the utility of new diagnostic methods, and alternative treatments have lead to large variations in practice patterns between centres. These national guidelines present evidence-based recommendations aiming at a uniform, safe and cost-efficient management of this large group of patients.

**Method:**

A working group of six experts with broad clinical and research experience was formed. Additional expertise from outside was consulted during the process. A national survey revealed significant variations in the management of patients with suspicion of appendicitis. The evidence provided in published guidelines and reviews were extracted and systematically graded, according to the GRADE methodology. This was supplemented by additional more recent and more directed search of the literature. Patients treated for appendicitis were involved through interviews. The guidelines were reviewed by external experts before the final version was determined.

**Results:**

The guidelines cover an extensive number of issues: pathology, epidemiology, aetiology, natural history, clinical and laboratory diagnosis, diagnostic scoring systems, diagnostic imaging, treatment, nursing care, follow-up, quality registers and quality indicators, among others. Special considerations related to children and pregnant women are covered.

**Conclusion:**

These national guidelines present an extensive and thorough review of the current knowledge base related to appendicitis, and provide up-to-date evidence-based recommendations for the management of this large group of patients.

## Introduction

**Fig. 1:**
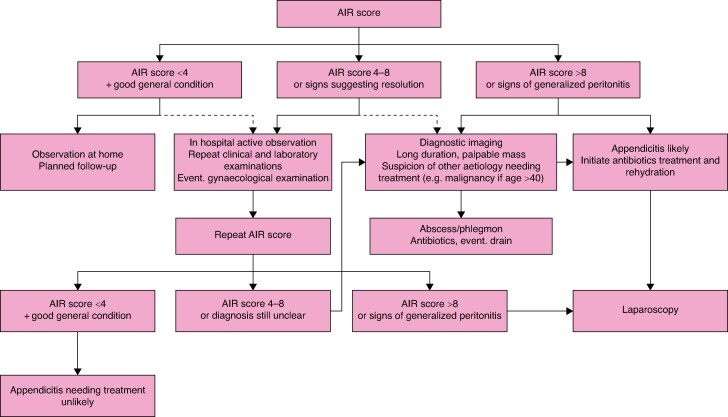
Algorithm for primary management of patients of all ages with suspected appendicitis, based on the Appendicitis Inflammatory Response (AIR) score

Appendicitis is a common condition at emergency departments in Sweden. The disease mainly affects younger patients but is seen in all age groups. About 11 000 cases of appendicitis are seen each year in our country. Mortality rates are very low but morbidity rates can be high in cases of perforated appendicitis. Although appendicitis has been traditionally regarded as a progressive condition that always requires rapid diagnosis and surgical treatment, in more recent years different approaches to this disease have emerged. Specifically, two types of appendicitis have been identified: complicated appendicitis, which should be detected and treated without delay, and uncomplicated appendicitis, for which management is currently controversial. In the latter, spontaneous resolution is common and non-operative management based on observation and/or antibiotic treatment has been advocated as an alternative to surgery. This diversified view on appendicitis motivates structured management based on risk stratification (*Fig. 1*).

These national guidelines are aimed at healthcare professionals who manage patients with suspected or confirmed acute appendicitis. The main objectives of these guidelines are to ensure equal quality of care for patients with appendicitis regardless of where they seek treatment; reduce morbidity and mortality rates in acute appendicitis; provide up-to-date evidence for the diagnosis and management of acute appendicitis; and make appendicitis care more patient-centred and effective. A summary of the recommendations is listed in *Table 1*.

**Table 1:** Summary of the evidence-graded recommendations in the Swedish national appendicitis guidelines

**Triage and diagnostics**
The decision whether the patient should be referred to an emergency department should be based primarily on history, clinical examination and CRP (⊕).
Scoring systems without acute blood tests seem promising to risk-stratify children in primary care for further management decisions (⊕).
When appendicitis is suspected, blood tests including leucocytes, neutrophils, and CRP should always be performed (⊕⊕⊕⊕).
An electrolyte panel should be made on admission, partly for possible correction before anaesthesia, and partly because hyponatraemia is a good marker of complicated disease, especially in children (⊕⊕).
Scoring systems provide objective support for assessing the likelihood of appendicitis and should always be used. They enable risk-stratified management for optimal resource utilization with fewer admissions, less use of imaging, and fewer operations (⊕⊕⊕⊕).
The AIR score has been shown in external validations to provide reproducible results for all ages and should be the first choice (⊕⊕⊕⊕).
Radiological diagnosis is particularly relevant in patients at intermediate risk of appendicitis (⊕⊕⊕⊕), where the diagnosis is unclear, or where other important differential diagnoses are likely (⊕⊕).
Ultrasound is the first choice, especially for examining children and pregnant women (⊕⊕).
Abdominal CT is the second choice, offering slightly higher specificity and sensitivity relative to ultrasound. However, it is associated with a small radiation risk, so there should be a clear indication for its use (⊕⊕).
Low-dose CT with contrast has similar accuracy as conventional CT (⊕⊕⊕⊕).
MRI has much lower availability and should only be used for pregnant women where ultrasound has been inconclusive (⊕⊕).
When appendicitis is suspected in pregnant women, blood tests including leucocytes, neutrophils, CRP, and a urine sample should always be taken (⊕⊕).
Ultrasound is the first choice in pregnant women (⊕), followed by MRI if available (⊕⊕⊕).
Observation with repeated blood tests and ultrasound in cases of suspected appendicitis is reasonable if the woman is not otherwise unwell. This does not appear to affect maternal or fetal outcome (⊕).
In pregnant women: if US is inconclusive or if US and MRI are not available or would significantly delay the diagnosis, CT may be indicated if there is a sufficiently strong suspicion of complicated appendicitis, unless the general condition is so affected that there is an indication for urgent surgery (⊕⊕).
Diagnostic laparoscopy seems safe in pregnant women when it comes to preterm birth and fetal loss at least in early pregnancy (<20 weeks). However, published evidence of the risks *versus* benefits is very limited and conflicting (⊕).
**Treatment**
A dose of broad-spectrum antibiotics should be given as prophylaxis for appendectomy (⊕⊕⊕⊕).
No postoperative antibiotics should be given in surgery for uncomplicated appendicitis (⊕⊕⊕⊕).
Postoperative antibiotics should be given after surgery for complicated appendicitis. The definitions of gangrenous and perforated appendicitis vary widely, making the studies difficult to interpret. There is probably no benefit for postoperative antibiotic treatment in gangrenous appendicitis (⊕⊕), but due to the uncertainty of the evidence, a 24-h postoperative treatment is recommended.
Postoperative antibiotics should be given after surgery for perforated appendicitis, especially if adequate source control has not been achieved. Start antibiotic treatment immediately upon suspicion and continue for 3–5 days after surgery (⊕⊕⊕⊕). Treatment can be switched to an oral option as soon as clinically feasible (⊕⊕⊕).
Thrombosis prophylaxis is not routinely recommended in surgery for acute appendicitis.
If the patient is over 40 years old and the operation lasts longer than 60 min, thrombosis prophylaxis should be considered (⊕).
Thrombosis prophylaxis should be administered to patients with clear risk factors for thrombosis, including pregnancy (⊕).
In assumed complicated appendicitis (not abscess and phlegmon), patients should be optimized and operated within 6 (–8) hours for the sake of well-being and to reduce the risk of complications (⊕⊕).
In assumed uncomplicated appendicitis, the risk of complications is not increased by up to 24 h from arrival at the emergency department to surgery (⊕⊕⊕⊕).
Laparoscopic appendectomy is recommended as the first choice for all patient groups and for both uncomplicated and complicated appendicitis (not abscess and phlegmon) (⊕⊕⊕⊕).
The standard three-port technique for laparoscopic appendectomy is recommended for both adults and children (⊕⊕).
Open appendectomy may need to be used in late pregnancy and in case of contraindication to laparoscopy, but it is always a matter of judgement in each case.
In complicated appendicitis with free fluid in the abdomen, aspiration of the fluid is recommended but not peritoneal lavage (⊕⊕⊕).
Postoperative abdominal drain is not recommended (⊕⊕⊕⊕).
Patients with appendicitis abscess should be treated primarily with intravenous antibiotics and drainage. Phlegmon should be treated primarily with intravenous antibiotics (⊕⊕⊕⊕).
Planned interval appendectomy after a conservatively treated abscess or phlegmon is not routinely recommended except in cases where malignancy is suspected and/or unclear findings at follow-up remain (⊕⊕).
Patients who have been treated conservatively for appendicitis abscess or phlegmon should be followed up with CT or colonoscopy (sometimes a combination of both) as there may be an underlying malignancy
In two randomized trials, antibiotic treatment was not significantly better than placebo and therefore cannot be recommended for the treatment of uncomplicated appendicitis (⊕⊕⊕⊕).
The ERAS concept speeds up recovery, shortens hospitalization time, and seems to lead to fewer reoperations (⊕⊕⊕).
Children who have undergone laparoscopic appendectomy for uncomplicated appendicitis can be discharged from hospital on the same day (⊕⊕).
Urinary catheters and central access are not indicated in children with complicated appendicitis (⊕⊕).
Pain relief for children at home usually consists of paracetamol and NSAIDs.
**Follow-up**
PAD should be made routinely in all patients (⊕⊕). If the resources are scarce all appendices in patients >40 years of age should undergo PAD to avoid missing an alternative diagnosis. To evaluate the quality of the clinic, all consecutive appendectomy specimens should be analysed at least for a period of time.
Follow-up is not necessary if PAD shows acute appendicitis.
Children do not need follow-up.
People under 40 years of age do not require follow-up unless their medical history suggests a malignant genesis.
People over 40 years of age are recommended to have a CT colonography or colonoscopy after 6–8 weeks to rule out malignancy (⊕). The findings on the abdominal CT scan can guide the selection of the most appropriate test.
Elective surgery after non-operative successful treatment is not recommended with the following exceptions (⊕⊕):
The patient has recurrent appendicitis symptoms (relapse)
Malignancy is suspected during follow-up.

CRP, C-reactive protein; CT, computed tomography; AIR, appendicitis inflammatory response; MRI, magnetic resonance imaging; US, ultrasound; ERAS, enhanced recovery after surgery; NSAIDs, non-steroidal anti-inflammatory drugs; PAD, pathologic anatomic diagnosis.

## Methods

### Working group

The working group consisted of representatives from all over Sweden who have broad clinical experience of acute appendicitis in different patient categories. The group also has extensive research experience in the field of appendicitis. Additional expertise outside the working group has been included throughout the process to reinforce specific parts of the guidelines (for example histopathology, choice of antibiotics, and radiology). Prior to publication, external experts reviewed the guidelines in a separate consultation round.

#### National survey

At the outset of this work, a questionnaire was distributed to all surgical clinics in Sweden (*n* = 60)^1^ and local guidelines for the management of appendicitis were collected. A total of 55 responses were collected, primarily from the heads of the departments. The survey clearly revealed significant variations in the management of patients with suspected appendicitis across hospitals. One consequence of this variability is the substantial differences in the reported incidence of appendicitis between regions. This highlights the urgent need for a more structured and standardized approach to the management of this patient group throughout the country.

### Guidelines development

The guidelines were developed in several steps, the first of which included the collection of already published major guidelines and reviews. The quality of these was assessed uniformly using the AGREE instrument^2^.

In 2020, a comprehensive international review was published, covering all important areas of acute appendicitis diagnosis and management and providing a thorough grading of the evidence^3^. The various parts of the guidelines have then been supplemented with additional more recent literature.

We followed the GRADE (Grading of Recommendations, Assessment, Development, and Evaluations)^4^ tool to grade the quality of the evidence of the recommendations as follows: *Strong scientific evidence* (⊕⊕⊕⊕), based on high- or medium-quality studies with no weakening factors in an overall assessment; *moderately strong scientific evidence* (⊕⊕⊕), based on high- or medium-quality studies with the presence of single weakening factors in an overall assessment; *limited scientific evidence* (⊕⊕), based on high- or medium-quality studies with weakening factors in an overall assessment; or *insufficient scientific evidence* (⊕), when scientific evidence is lacking, the available studies are of low quality, or studies of similar quality are contradictory.

Specific literature searches, with the help of a specially educated librarian, have been carried out in some cases where the evidence was limited, such as suspected appendicitis in primary care.

The final version was accepted after multiple rounds of consultation with the National Collaborative Group on Pharmaceuticals and Medical Technology, the National Working Group on Pharmaceuticals and Knowledge Support, and all national surgical clinics and surgical societies, and a last consultation with special emphasis on the organizational and financial consequences of the guidelines for final approval by the National Program Area for Gastrointestinal Diseases.

#### Patient involvement

Several types of patients treated for acute appendicitis (both adult, paediatric, and pregnant) were involved through interviews. This knowledge, together with literature searches, was mainly used in the development of quality indicators.

#### Limitations of current literature

The literature on appendicitis is extensive and difficult to penetrate. Results can be greatly influenced by patient selection as well as the diagnostic criteria and the outcome measures used.

Reliable assessment of the negative appendectomy rate and of the performance of diagnostic variables requires histopathological examination of all resected specimens with accepted diagnostic criteria, that is transmural invasion of neutrophils (gold standard). In the absence of histopathological diagnosis, the perioperative assessment may be accepted, but this varies greatly between operators^5,6^. For the non-operated patients, a follow-up is crucial to identify those who will eventually need surgical treatment.

The diagnostic value of variables is influenced by patient selection and the prevalence of the disease in the study population. Studies on unselected patients examined for suspected appendicitis are more representative and more reliable than studies based on only operated patients. Because spontaneously resolving appendicitis appears to be common, studies reporting results for complicated appendicitis are more reliable in assessing the diagnosis of appendicitis requiring surgery.

When studying small patient groups with a relatively low incidence of appendicitis, it is difficult to conduct prospective studies. This applies, for example, to children under 5 years of age and pregnant women. To overcome this difficulty, healthy controls can be used as a comparison.

Given the current state of knowledge, some studies are not feasible from an ethical perspective. There will therefore be areas and recommendations in the guidelines that, despite a crucial role in clinical practice, can never receive the highest level of evidence.

For continuous variables, such as concentration of leucocytes or C-reactive peptide (CRP), or radiological measurement of appendix diameter, an overlap between sick and healthy patients is usually seen. Often, sensitivity and specificity are reported at only one cut-off value. These diagnostic values rarely become useful. More useful are those studies that specify two measurement points—one for high sensitivity and one for high specificity. The overall discriminatory value can also be compared with receiver operating characteristic (ROC) curves.

The proportion of perforations is difficult to use as a quality indicator, as the size of the denominator (total number of appendicitis cases) can be affected by differences in the detection and treatment of mild appendicitis that may otherwise heal spontaneously and be undiagnosed. Therefore, to accurately assess the impact of an intervention, both the number of perforations and the total number of appendicitis cases before and after the intervention should be reported.

## Background and causes

### Pathology and classification

**Summary**

1. Histological examination of the removed appendix (Pathologic Anatomic Diagnosis (PAD)) is the gold standard for the diagnosis of acute appendicitis. For non-operated patients, the diagnosis may be based on imaging results. However, there is no generally accepted consensus.
2. Criteria for the histological diagnosis are transmural inflammation, with the presence of white blood cells (granulocytes) in all wall layers of the appendix. However, pathologists apply different criteria.
3. The severity of the disease, based on histological, intraoperative and radiological findings, is classified as:
  Uncomplicated: phlegmonous
  Complicated:
    Gangrenous: disintegration of the deeper wall layer of the appendix.
    Perforated: tissue breakdown with passage of intestinal content into the abdominal cavity, or abscess.
    Phlegmon: the inflamed appendix is surrounded by secondarily inflamed omentum.

The appendix is a true diverticulum, about 5–15 cm long, which originates from the first part of the large intestine (caecum). Acute appendicitis represents an acute inflammation of the appendix. The diagnosis can be made based on radiological, clinical, and intraoperative findings and the histopathological examination of the removed appendix. In most cases, several modalities are combined for diagnosis and there is no consensus.

The histopathological classification is considered the gold standard. However, pathologists apply different nomenclature and criteria, which may affect the assessment of the proportion of operated patients where the appendix is not inflamed (so-called negative appendectomy)^7–11^. Uncomplicated phlegmonous appendicitis is characterized by invasion of inflammatory cells past the mucosa and submucosa into the muscularis propria (transmural inflammation). In complicated appendicitis there is necrosis of the appendix tissue (gangrenous appendicitis) which may lead to breakdown of the appendix wall and free passage of intestinal contents to the abdominal cavity (perforated appendicitis)^11^. However, clinical (see Diagnosis) and radiological (see Imaging) signs of perforation may not always be detected on histopathological examination. A diagnosis of perforated appendicitis is therefore usually based on perioperative findings of pus in the abdomen, but free pus in the abdomen may also be caused by the inflammatory reaction per se and does not always contain bacteria.

According to the Swedish Society of Pathology, a histopathological report should always include information on granulocyte infiltration in the different layers including transmural inflammation. Wall necrosis should be indicated. Perforation can sometimes be demonstrated, but intestinal contents on serosal surfaces are usually accepted as indirect evidence. Perforation or not should be commented on.

Acute appendicitis may be covered by omentum, fibrin, or other tissue, which prevents free perforation to the peritoneal cavity and creates a palpable appendiceal mass. Based on radiological findings, this mass is classified as appendicitis abscess if it contains fluid/pus, or appendicitis phlegmon if it contains only inflamed tissue.

Chronic appendicitis is a rare and controversial condition, clinically characterized by persistent, mild abdominal pain that can last for an extended period although inflammatory markers often remain within normal ranges^12–14^. In retrospective studies, about three-quarters of these patients become pain-free after appendectomy. Histopathological examination may reveal granulomatous inflammation with fibrosis, and sometimes crypt abscesses. However, these changes can also be observed in appendix specimens from asymptomatic patients^7,11,13^. In a randomized trial including patients with persistent or recurrent right lower-quadrant abdominal pain, laparoscopic appendectomy was associated with reduced pain at 6 months after surgery compared to laparoscopic inspection only. However, no association was found between freedom from symptoms and histology^14^.

### Diagnosis codes

Administrative health records use diagnosis codes according to the International Classification of Diseases (ICD) coding system. Several versions have been published and the definitions have varied over time, which has affected the interpretation of longitudinal comparisons. Especially after the latest revision in 2010, very large variations have arisen between reporting hospitals^15^. See further under Quality registers and quality indicators.

Current ICD-10 codes for appendicitis are: C35.2 Acute appendicitis with generalized peritonitis, C35.3 Acute appendicitis with localized peritonitis, C35.8 Other and unspecified acute appendicitis, C36.9 Other appendicitis, and K37.9 Unspecified appendicitis.

### Epidemiology

**Summary**

1. Acute appendicitis is common with an incidence of about 120/100 000 inhabitants/year, corresponding to just over 11 000 cases/year in Sweden.
2. There is a peak in incidence between about 10 and 25 years of age.
3. Incidence is decreasing in children, but is stable in adults in Sweden.
4. Acute appendicitis is associated with very low mortality rates.

**Fig. 2:**
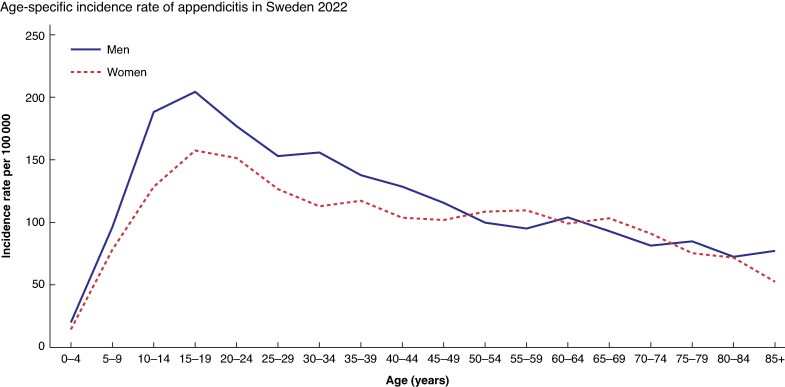
**Incidence of acute appendicitis (International Classification of Diseases K35–K37)/100 000 inhabitants in men and women at different ages in Sweden 2022**Source: Statistics and diagnoses in in-patient and specialized open care [internet]. Stockholm: Socialstyrelsen. Available at: https://www.socialstyrelsen.se/statistik-och-data/statistik/statistikdatabasen/.

In Sweden, the incidence of diagnosed appendicitis is about 120/100 000 inhabitants/year, with a higher incidence in men. Appendicitis is relatively uncommon in children under 5 years of age, peaking in adolescence and then declining (*Fig. 2*).

The incidence of complicated and uncomplicated appendicitis shows different patterns in many studies. The lockdown period during the coronavirus disease-2019 (COVID-19) epidemic was associated with large variations in the incidence of uncomplicated appendicitis in relation to age, geographical areas, and over time, in contrast to complicated appendicitis, which remained almost the same regardless of these factors^16–22^.

**Fig. 3:**
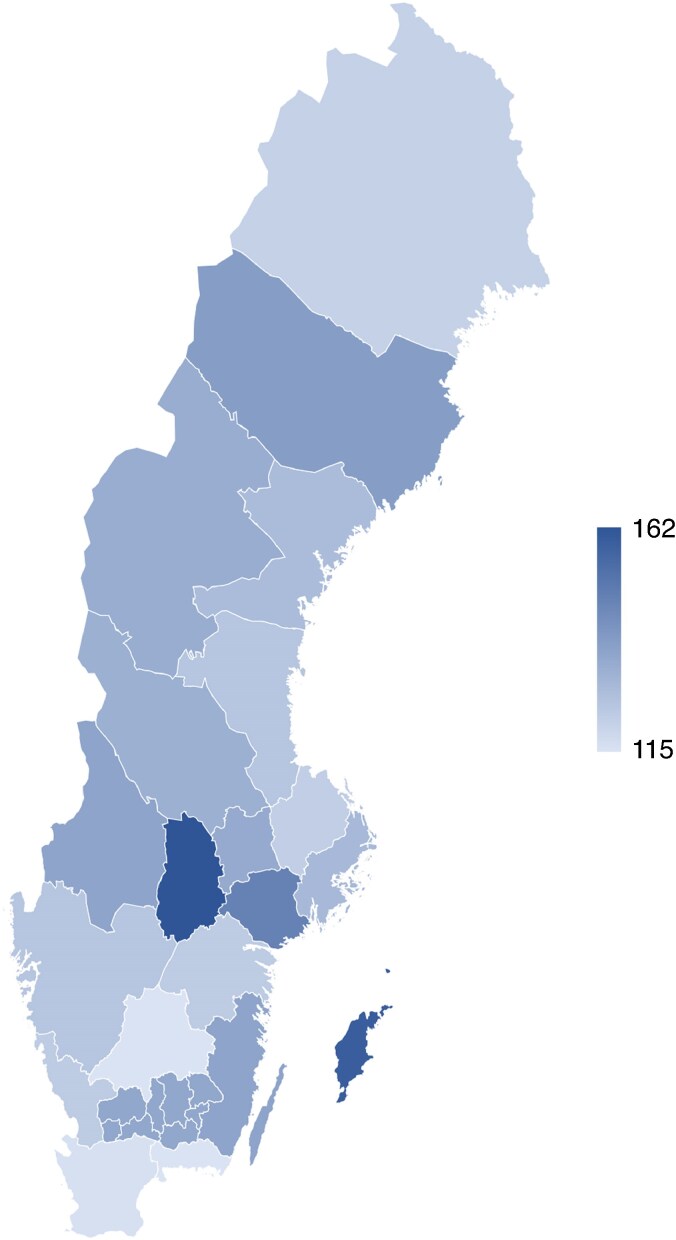
**Incidence of acute appendicitis in the different regions in Sweden 2018–2022 (number of patients/100 000 inhabitants)**Source: Statistics and diagnoses in in-patient and specialised open care [internet]. Stockholm: Socialstyrelsen. Available at: https://www.socialstyrelsen.se/statistik-och-data/statistik/statistikdatabasen/.

In Sweden, the incidence has developed differently for children and adults. In children, a declining incidence is seen, mainly of uncomplicated appendicitis^19^. In adults, a corresponding declining incidence has been seen up to a lowest level of 85/100 000 inhabitants in 2004, after which, unlike in children, it has risen and plateaued at around 120/100 000 inhabitants. The reason for these variations is unclear. There are also geographical variations in incidence within Sweden, which most likely reflect differences in the management of patients with suspected appendicitis (*Fig. 3*).

The mortality rate from appendicitis is very low in high-income countries (0.09–0.24%) compared to low-income countries (1–4%)^23^. According to the National Board of Health and Welfare, 12 patients died due to appendicitis in 2020 in Sweden (0.12/100 000 inhabitants), all of whom were over 65 years of age. The same number of annual deaths was seen in a chart review of patients who died in Sweden within 30 days of appendectomy between 1987 and 1996^24^. Co-morbidities and age are crucial contributors to mortality, not least in the case of negative appendectomy, as the patient is exposed to an unnecessary burden and the underlying diagnosis may be missed^25^. Mortality is present in all stages of disease but is doubled in perforated compared to non-perforated appendicitis and in negative appendectomy (likely due to the underlying undetected morbidity). Mortality rate is low in younger patients but increases in those over 75 years of age^24–26^. In pregnant women, mortality from appendicitis is extremely rare but not absent^27^.

### Aetiology and heredity

**Summary**

1. The aetiology of appendicitis is multifactorial, with obstruction, environment, gender, age, and genetic and immunological factors being important (⊕⊕⊕).

The aetiology of appendicitis is not completely understood. Complicated and uncomplicated appendicitis have different associations with multiple factors. Uncomplicated appendicitis varies geographically, between different ages and different ethnicities, and over time. This pattern is not seen for complicated appendicitis^17^. There are also differences in association with genetic factors^28–31^. This suggests that these represent two different entities.

Several factors are involved in the pathogenesis^11^. Obstruction of the appendix lumen, usually due to a calcified faecal stone, may be the cause especially in cases where a perforation develops^32–34^. However, faecal stones can be seen in healthy people without symptoms^35^. Large faecal stones are associated with complicated appendicitis^36^. Local outbreaks, space–time clusters and seasonal variation may suggest an infectious genesis, but no specific agents have been identified^37–39^.

The inflammatory response of the immune system seems to be important. Children who develop allergy have a lower risk of later developing complicated appendicitis compared to non-allergic children^40–42^. Patients who have had surgery for appendicitis before the age of 20 are less likely to develop ulcerative colitis^43,44^. This relationship is also seen within families, suggesting that appendicitis and ulcerative colitis have common, but opposite, underlying genetic factors^45^. Pregnancy has a protective effect during the first and especially the third trimester^46^.

Several reports have shown an increased risk of appendicitis within families and among relatives, suggesting genetic factors^31,47–55^. However, several of these studies have methodological weaknesses, especially because they often study the risk of appendectomy, which may be influenced by social factors^56,57^. Speculatively, it is possible that parents who have experienced appendicitis themselves may be more likely to seek medical care for their children if they develop abdominal pain, leading to biased care-seeking behaviour. In addition, data in the studies above have often been collected retrospectively through questionnaires, which can cause recall bias. A twin study, using data from the Swedish Patient Registry and the Swedish Twin Registry, could not identify a genetic effect, but found a significant influence of environmental factors^51^. However, some studies have shown association with specific genes^31,55,58^.

In summary, appendicitis has a complex aetiology, where infection, obstruction, environment, gender, age, and genetic and immunological factors are involved.

### Natural history

**Summary**

1. Circumstantial evidence suggests two different types of appendicitis—one progressive to perforation and one spontaneously healing, possibly recurrent (⊕⊕⊕).
2. In most cases, perforation is likely to occur early, before arrival at the hospital (⊕⊕).

Knowing its natural history is of great importance for the optimal management of a disease. Indirect evidence suggests that complicated and uncomplicated appendicitis represent two different types of disease with different natural history—a type that can rapidly progress to gangrene and perforation, or circumscribed abscess, and a type that is spontaneously resolving and possibly recurrent^59^.

The spontaneous resolving form can often go undiagnosed. A meta-analysis of four studies that randomized patients with acute non-specific abdominal pain to laparoscopy or observation showed a six-fold higher rate of diagnosed appendicitis in the laparoscopy group suggesting spontaneous resolution in the other^60–64^. A systematic review and meta-analysis of the impact of reduced access to healthcare during the COVID-19 pandemic in 63 reports showed a reduced incidence of uncomplicated appendicitis during the pandemic but no impact on complicated appendicitis^22^. In two placebo-controlled trials of patients with presumed uncomplicated appendicitis, over 90% and 80% healed, respectively, irrespective of whether they received antibiotics or placebo, showing that spontaneous resolution is common in uncomplicated appendicitis^65,66^. More than 60% of 182 patients with ultrasound-verified low-grade appendicitis who received supportive care only were still cured after 9 years of follow-up^67^. Increased use of imaging is associated with an increased incidence of diagnosed and treated appendicitis. In two studies that randomized patients to CT or observation, there was a significantly higher number of patients diagnosed with appendicitis in the CT group^68,69^.

There is a clear correlation between the duration of symptoms before arrival at the hospital and the proportion of perforations, with a higher proportion of perforations when the duration of symptoms is long^70–72^. However, there is no increase in the proportion of perforations when treatment is delayed for up to 24 h after arrival at the hospital^73–80^. This may indicate that perforation in most cases has already occurred when the patient seeks care or may occur shortly after arrival at the hospital for patients with a short duration of symptoms. Due to spontaneous resolution of uncomplicated appendicitis, increasing duration of symptoms on arrival will result in the selection of those with complicated disease. Selection due to spontaneous resolution may therefore be a possible explanation for the increasing proportion of complicated disease with increasing duration of symptoms^16,59^.

The proportion of perforations is a composite measure that is dependent on both the number of perforations (numerator) and the total number of appendicitis cases (denominator). Many studies comment only on changes in the proportion of perforations after an intervention, although the change is often only seen in the number of appendicitis cases (denominator) whereas the number of perforated appendicitis cases in the numerator may be unchanged. An increased proportion of perforations can therefore often be seen in association with a decreased total number of appendicitis cases.

For example, the introduction of CT diagnostics showed a sharp decrease in the rate of perforation^81^, but careful analysis shows that this is explained by an increase in the number of patients treated for non-perforated appendicitis.

The optimal pace of management of patients with suspected appendicitis depends on the nature of the underlying disease. The focus in the past has been to prevent perforation by prompt surgery in all patients with suspected appendicitis, even if associated with a high rate of negative appendectomies. If perforation typically occurs before hospital arrival, the focus should be on promptly diagnosing and surgically treating patients with progressive or complicated appendicitis. In contrast, the need for rapid diagnosis and treatment is less urgent for patients with suspected uncomplicated appendicitis. This does not mean that these patients may not require equally rapid assessment for symptomatic treatment, such as analgesia. There are currently no reliable ways to predict the prognosis of patients with suspected uncomplicated appendicitis^82^. Many will resolve, but some will not heal spontaneously and may finally require surgical treatment.

## Primary prevention and screening

As no risk factors for appendicitis are known, there are no preventive measures. Screening has no role in predicting appendicitis. Nor is appendicitis or appendectomy associated with the risk of future malignancy or other morbidity^83^.

## Diagnosis

### Triage

Patients presenting with acute abdominal pain represent a heterogeneous group with varying probabilities of appendicitis and severity of disease. In adult patients, the proportion diagnosed with appendicitis ranges from approximately 2.5% to 28%, depending on the level of care^84–88^. In children with acute abdominal pain this figure is considerably lower, estimated at around 1–3%, and generally only few children with acute abdominal pain require surgery^89,90^. It is therefore reasonable that children with acute abdominal pain in the paediatric emergency department, especially preschoolers and infants, should be examined by a paediatrician in the first instance. Overall, the investigation and pace of care should be tailored to each patient, also depending on the level of care the patient is encountered at, for example the telephone helpline, health centre, or emergency department.

This justifies the use of triage and risk-stratified management based on assessment of medical history, symptoms, clinical examination, and basic laboratory tests. For a more objective risk assessment, a clinical score can be used to support decision-making^91,92^. For the management of patients with abdominal pain and possible appendicitis the top priority is to identify patients with a high probability of complicated appendicitis for rapid diagnosis and optimization before surgery. A second priority is to identify those patients with a very low probability of appendicitis who may be managed by home observation with a planned return visit. The remaining patients can then be more effectively assessed with a higher accuracy.

#### Assessment by a gynaecologist

For women with acute abdominal pain where a gynaecological differential diagnosis is reasonably suspected, such as ovarian torsion, salpingitis, or endometriosis, contact should be made with a gynaecologist for gynaecological examination including vaginal ultrasound. A pregnancy test should be performed in women of childbearing age with acute abdominal pain. Extrauterine pregnancy with haemorrhagic rupture of a fallopian tube and blood in the abdomen can cause generalized abdominal pain, peritoneal irritation, and severe upper abdominal pain in case of large amounts of blood in the abdominal cavity.

#### Suspected appendicitis in the primary care setting

**Recommendation**

1. The decision whether the patient should be referred to an emergency department should be based primarily on history, clinical examination, and CRP (⊕).
2. Scoring systems without acute blood tests seem promising to risk-stratify children in primary care for further management decisions (⊕).

Abdominal pain in primary care is common, but Swedish data on how many patients with appendicitis initially present to primary care are lacking and extrapolation from other countries can be difficult as healthcare systems including primary care often differ significantly. A study from Norway showed that about 25% of primary care patients with acute abdominal pain are referred to the emergency department, of whom about 50% have appendicitis^93^. In a study from the Netherlands, about 16% of all children who presented with acute abdominal pain in primary care were referred, of which about a third were subsequently diagnosed with appendicitis^94^. In the same study, primary care assessment missed about 20% of children with appendicitis at their first presentation. However, this is not different from the precision of an emergency department.

Diagnosis is more difficult in primary care as the population is unselected and the prevalence of appendicitis is low, especially in children, women of childbearing age, and the elderly. This means that the predictive value of clinical and laboratory tests is reduced. In addition, there is often no possibility to urgently analyse leucocytes and neutrophils. Given the limited evidence for triage and assessment of suspected appendicitis in primary care, published recommendations may be based on those for emergency departments, despite the higher prevalence of appendicitis there, with better results from scoring systems and clinical judgement based on history, status, and laboratory examinations. For best results, history and clinical examination should be combined with examination of leucocytes, neutrophils, CRP, and urinary stick. These results can then be summarized in a clinical score (see Scoring systems) that can guide the further management, including possible referral to an emergency department.

If analysis of leucocytes and neutrophils is not possible, the history and a thorough clinical abdominal examination, together with CRP and urinary stick results, may form the basis for the decision to refer. The focus should then be on assessment of the patient’s general condition and clinical findings (palpation tenderness, rebound tenderness, and abdominal muscle guarding), which are more reliable than reported symptoms^94^. A recently published large retrospective study has shown promising results in children with a purely clinical scoring system without blood tests, as a tool to help risk-stratify patients^95^.

A positive urinary dipstick (often ery+ and/or leu+) does not exclude appendicitis but, on the contrary, can be seen in about 40% of patients, sometimes leading to differential diagnosis problems.

Pregnant women of viable gestational age (from gestational week 22 + 0) with abdominal pain should be assessed in the labour ward or an obstetric clinic. For women in early pregnancy, consider pregnancy-related conditions, especially extrauterine pregnancy, and refer to an emergency department or gynaecological emergency department.

### Symptoms

Acute appendicitis usually begins with malaise and moderate central abdominal pain or with a diffuse location. The functions of the gastrointestinal tract are affected, which may cause loss of appetite, nausea, bloating, or vomiting^96–99^.

Later, usually within a few hours, there is local irritation of the peritoneum near the appendix, resulting in more localized pain and tenderness in the lower-right quadrant of the abdomen, as well as pain on coughing and body movement. This shift in the localization of pain and tenderness, or pain migration, is a classic symptom.

The initial general and diffuse abdominal pain comes from the innervation of the appendix or intestines surrounding the appendix. The later localized pain is due to progression of the inflammation in the appendix leading to peritoneal irritation^100^. At this stage, fever usually occurs.

With perforation, which can occur within a few hours after the onset of symptoms, the general condition is more severely affected, with increased fever and widespread and intense pain. In the case of perforation into the free peritoneal cavity, there is a generalized irritation of the peritoneum with increased abdominal pain at movements, such as coughing, jumping, or ‘heel down’ (when the heel is dropped to the floor from standing on toes). The inflammation can sometimes be localized to the small pelvis and then cause irritation of the bowel with painful rectal contractions and frequent bowel movements.

If appendicitis is suspected with symptoms lasting more than 3 days, accompanied by signs of an inflammatory response and peritonitis, complicated appendicitis should be suspected.

It is important to recognize that individual symptoms have low diagnostic value. Painful walking and pain on coughing and movement are important symptoms but have relatively low discriminatory and predictive values^97,99^. One reason is that the interpretation of the patient’s medical history and examination findings is subjective, and varies greatly between examiners^101,102^.

### Clinical findings

#### General condition

The general condition is often affected with malaise and fever. High fever may be seen in complicated appendicitis with free peritonitis^96,99^.

#### Findings at the abdominal examination

Early on, the patient reports diffuse pain and tenderness on palpation of the abdomen. Later on, the pain and tenderness is localized to the lower-right quadrant (McBurney’s point). However, the localization of pain and tenderness may be influenced by the anatomical position of the appendix in the abdomen^103^. Signs of peritoneal irritation may be attenuated or absent if the appendix is located behind the colon or in the lesser pelvis.

Percussion tenderness, involuntary muscle contraction on abdominal palpation, and direct and indirect rebound tenderness are indications of peritoneal irritation. These findings are the most reliable in suspected appendicitis^96,97,99^. However, the interpretation of clinical findings on abdominal examination is a subjective process, which explains the wide variation between examiners^102^.

An appendiceal abscess and a phlegmon (accumulation of omentum surrounding the inflammation) can produce a palpable mass in the right quadrant (appendiceal mass). Unlike an abscess, the phlegmon usually contains no drainable pus. If a palpable mass is found, the possibility of an underlying malignancy should be considered, especially in elderly patients^104^. An abscess in the pelvic pouch can sometimes be felt as a tender bulge on rectal examination. However, routine rectal examination has no value in the diagnosis of appendicitis^105^. In children, the rectal examination should only be performed in exceptional cases.

### Children

Appendicitis has a relatively similar clinical presentation in children and adults^104^. The main differences are in the youngest children^106–111^. Compared to an adult patient, the history may be uncertain, including the duration of symptoms. Decreased appetite and vomiting are common in younger children with conditions unrelated to abdominal issues. Abdominal status, such as suspected peritonitis, is also more difficult to assess in these patients.

The classic constellation of appendicitis symptoms has a relatively low predictive value in younger children. Many of the symptoms are either missing or unspecific. Important symptoms in children with suspected appendicitis are painful walking, vomiting, and pain on percussion and palpation of the abdomen. It is important to consider the diagnosis of appendicitis even in the youngest children (under 5 years old), despite its much lower incidence in this age group.

The younger the child is, the more diffuse the symptoms are, partly because of the difficulties with history and status in this age group and partly because a larger proportion have complicated appendicitis, which paradoxically can produce symptoms that further complicate the diagnosis (for example diarrhoea)^108–111^. The youngest children also seek treatment much later in the process^108–111^. This is also the group where misdiagnosis is most common. In these children, extra-abdominal causes of what is triaged as acute abdomen are also more common. The examination should therefore include a complete status including lungs, mouth, throat, ears, skin, external genitalia, and hips^99,108–111^.

### Differential diagnoses

Acute appendicitis is a possible differential diagnosis in virtually all cases of acute abdominal pain. Gender and age, which influence the prevalence of different acute abdominal conditions, should be taken into account in the clinical assessment.

The most common differential diagnoses of acute appendicitis are:

1. Surgical: perforated ulcer, acute cholecystitis, pancreatitis, intestinal obstruction, invagination, volvulus, inflammatory bowel disease, irritable bowel syndrome, diverticulitis, malignancy, incarcerated inguinal hernia.
2. Non-surgical: gastroenteritis, glandular abdomen/lymphadenitis, constipation.
3. Urological: right-sided kidney stones, ureteral stones or pyelonephritis, urinary tract infection, urinary retention, testicular torsion (especially in boys/younger men).
4. Gynaecological: extrauterine pregnancy, ovarian torsion or rupture of ovarian cyst, haemorrhagic corpus luteum, salpingitis, endometritis or tubo-ovarian abscess, endometriosis, myoma necrosis, dysmenorrhoea.
5. Others: basal pneumonia, lumbago, ketoacidosis, acute porphyria, Mediterranean fever.
6. In children: constipation, gastroenteritis, mesenteric lymphadenitis, terminal ileitis, inflamed Meckel’s diverticulum, lower respiratory tract infection, infected urachus cyst.
7. In young adults and adolescents: mesenteric lymphadenitis, terminal ileitis, Crohn’s disease, ovarian torsion, testicular torsion.
8. In the middle-aged and elderly: cholecystitis, diverticulitis, ureteral stones, abdominal malignancy.

### Laboratory investigations

**Recommendation**

1. When appendicitis is suspected, blood tests including leucocytes, neutrophils, and CRP should always be taken (⊕⊕⊕⊕).
2. An electrolyte panel should be made on admission, partly for possible correction before anaesthesia, and partly because hyponatraemia is a good marker of complicated disease, especially in children (⊕⊕).

Appendicitis causes a general inflammatory reaction with fever and increased inflammatory markers in the blood. Several biochemical markers have been reported, but the most widely used are leucocytosis, neutrophilia, CRP elevation and lymphopaenia. Lymphopaenia can be seen especially in complicated appendicitis^112,113^. A low concentration of leucocytes can sometimes be caused by a lymphopaenia, so the proportion of neutrophils or the neutrophil/lymphocyte ratio has a stronger diagnostic value than the concentration of the individual forms, especially in complicated appendicitis^97,113–115^.

The inflammatory response is continuous from mild inflammation, associated with a low probability of appendicitis, to severe inflammation, indicating a high probability. Two cut-off points, representing high sensitivity and high specificity, should therefore be used for optimal utilization of their diagnostic value.

These inflammatory markers have a strong diagnostic value that exceeds findings at clinical examination^3,115–117^. Their discriminatory ability increases greatly when combined, which is facilitated by their inclusion in a clinical score^2^. When all inflammatory markers are completely normal, appendicitis, especially complicated appendicitis, is very unlikely^97,118,119^. Therefore, results of these laboratory investigations should always be available already at the primary assessment of patients with suspected appendicitis.

The inflammatory response is dynamic so repeated examinations of all these variables is warranted in unclear cases^115^. Leucocytes react quickly while CRP starts to rise after 12–24 h, reaching its highest level within 48 h. Conversely, CRP declines with a delay and may remain elevated for 24 h after other signs indicate resolution of inflammation^120^.

Other recently recognized markers include hyponatraemia, which appears to be a predictor of complicated disease, particularly in children^121–124^.

In summary, the recommended tests to assess a patient with suspected appendicitis are:

- leucocytes
- neutrophils (with calculation of the proportion of neutrophils)
- CRP
- electrolyte status
- urinary stick for U-hCG in women of childbearing age.

Additional tests may be required to exclude differential diagnoses and depending on the patient’s underlying and current medical condition.

#### Children

As in adults, no single blood test has sufficient sensitivity or specificity to diagnose acute appendicitis. However, combination of several inflammatory markers provides high predictive values^99,108,116^, especially when combined in a clinical score^125^.

The leucocyte concentration is normally higher in young children, which should be taken into account^126–128^. Values above 15 × 10^9^/l and below 8.5 × 10^9^/l significantly increase and decrease the likelihood of appendicitis respectively, but neutrophils must also be assessed. Children are more likely to have a reduction in the number of leucocytes in progressive inflammation, resulting in an increasing proportion of neutrophils. A commonly used cut-off value in the form of an absolute neutrophil count below about 7 × 10^9^/l has been shown in well-designed studies to exclude appendicitis with relative confidence. CRP reacts with a 12–24 h delay. A normal CRP should therefore be taken with caution in patients with short duration of symptoms. If symptoms last more than 12 h, a normal CRP halves the risk of the child having acute appendicitis^99,108,116^ and the diagnostic value of CRP increases with time^129^.

Hyponatraemia (<136 mmol/l) has been shown in recent studies in children to be a good predictor of complicated appendicitis^121,123,124^.

### Observation with repeat examination

In patients with suspected appendicitis without signs of complicated appendicitis or other disease requiring immediate surgical treatment, observation with repeat examination (abdominal palpation and inflammatory markers) may lead to an improved diagnosis and fewer surgeries for uncomplicated appendicitis indicating spontaneous resolution^115,130–133^.

Routinely performed imaging was compared with observation with repeated examinations and selective imaging in two randomized studies of patients with intermediate likelihood of appendicitis. Observation and repeat examination resulted in not only fewer radiological examinations but also fewer operations for uncomplicated appendicitis. It also resulted in the same risk of perforation and the same proportion of negative explorations compared with routine immediate imaging^67,68^. Similar results were seen in another randomized trial comparing selective with routine use of imaging in patients with acute abdominal pain^134^.

### Scoring systems

**Recommendation**

1. Scoring systems provide objective support for assessing the likelihood of appendicitis and should always be used. This strategy enables risk-stratified management for optimal resource utilization with fewer admissions, less use of imaging, and fewer operations (⊕⊕⊕⊕).
2. The AIR (Appendicitis Inflammatory Response) score has been shown to provide reproducible results for all ages and should be the first choice (⊕⊕⊕⊕).

Clinical diagnosis is based on a large number of indicators that are combined to assess the likelihood of appendicitis. The subjective evaluation of the importance of symptoms and clinical findings varies widely between examiners^102,135^. Clinical scoring systems can be used to help make this process more objective. They are based on variables that have been shown to have high diagnostic value and are given risk weights based on a large number of patients. To reduce variability in the subjective assessment, objective variables such as temperature and inflammatory markers are given more focus and the intensity of subjective variables like symptoms and clinical findings is graded.

The use of a clinical score as a stratification tool may lead to better resource utilization. Three prospective interventional studies^68,69,136^ resulted in fewer non-productive admissions, reduced use of imaging, and fewer operations when scores were utilized. One study found a reduced use of imaging in low-risk patients according to the paediatric Appendicitis Risk Calculator (pARC)^137^. Risk-stratified algorithms based on scoring systems are therefore recommended for optimal resource utilization^3,68,92,111^. Scoring systems also have value for objectively describing and comparing patients in the research context.

Many scoring systems have been developed. A bibliometric analysis identified 74 of them^138^. The Alvarado score had the most citations, followed by the AIR score. Unfortunately, many scoring systems have been evaluated retrospectively only on operated patients. Only a few have undergone a comparative prospective external validation on patients with suspected appendicitis.

The AIR score (*Table 2*), constructed in a Swedish context, has been recommended in three major reviews comparing a large number of scoring systems^3,139–141^. The AIR score outperformed the Alvarado score in nine studies^141–149^, the Adult Appendicitis Score in one study^150^, and a large number of scoring systems for men^139^. The AIR score has proved to work well in children^125,143,145^, where it demonstrated better results than both the paediatric Appendicitis Score and the Alvarado score^145^.

**Table 2:** Appendicitis inflammatory response (AIR) score

Parameters	Points
Vomiting	1
Pain in right iliac fossa	1
**Rebound tenderness/abdominal muscle defence**	
Slight	1
Moderate	2
Strong	3
Temperature ≥ 38.5°C	1
**Leucocyte concentration**	
10–14 × 10⁹/l	1
≥15 × 10⁹/l	2
**Proportion of neutrophils**	
70–84%	1
≥85%	2
**CRP concentration**	
10–49 mg/l	1
≥50 mg/l	2
Total score	12

High probability (AIR score > 8): Patients with suspected appendicitis with signs of peritoneal irritation and an inflammatory response are highly likely to have appendicitis, motivating surgical exploration. Diverticulitis may be a differential diagnosis in the elderly. Low probability (AIR score < 4): Patients with suspected appendicitis without signs of peritoneal irritation and inflammatory response and with an unaffected general condition have a low probability of appendicitis. Observation at home with a planned follow-up the next day or earlier if deterioration occurs, is often safe. Intermediate probability (AIR score 4–8): Active observation in hospital with repeated laboratory and clinical examination after 4–8 h is recommended in this group. If the diagnosis remains unclear after observation, additional investigations such as gynaecological consultation, ultrasound, or computed tomography (and possibly diagnostic laparoscopy) may be indicated. CRP, C-reactive peptide.

#### Stratified management based on the AIR score

A diagram for stratified management of patients with suspected appendicitis based on the AIR score is shown in *Fig. 1*. If the AIR score is less than 4, complicated appendicitis is very unlikely (negative predictive value 99%). In this often quite large group, observation at home may be considered with planned follow-up the next day by telephone or a return visit if there is no clinical improvement, or earlier if there is deterioration. With an AIR score above 8, the positive predictive value for appendicitis is 86%, and particularly high for children under 15 years of age (96%)^125^. With this high likelihood of appendicitis, diagnostic laparoscopy may be considered as the next course of action, as further diagnostic investigation can rarely rule out appendicitis^3,151^. However, imaging may be indicated if the duration of symptoms exceeds 3 days or if an abscess or phlegmon is suspected, as these may need conservative treatment. Imaging may also be indicated in patients over 40 years of age where diverticulitis may be a common differential diagnosis and there may be an underlying malignancy^152,153^.

## Diagnostic imaging

**Recommendation**

1. Radiological diagnosis is particularly relevant in patients at intermediate risk of appendicitis (⊕⊕⊕⊕), where the diagnosis is unclear, or other important differential diagnoses are likely (⊕⊕).
2. Ultrasound is the first choice, especially for examining children and pregnant women (⊕⊕).
3. Abdominal CT is the second choice, has slightly higher specificity and sensitivity but is associated with radiation risk, even if it is small, so there should be a clear indication for examination (⊕⊕).
4. Low-dose CT with contrast has similar accuracy as conventional CT (⊕⊕⊕⊕).
5. MRI has much lower availability and should only be used for pregnant women where ultrasound has been inconclusive (⊕⊕).

Diagnostic imaging with ultrasound (US), CT and, in specific cases, MRI is of great importance for the diagnosis of appendicitis but also for differential diagnosis, especially in patients with a medium probability of appendicitis, in patients >40 years of age because of the slightly increased risk of malignancy, and in patients with symptom duration >3 days because of the increased risk of abscess.

However, the role of imaging in suspected appendicitis represents a controversial topic internationally^3,154^. In patients with a high probability of appendicitis, imaging can rarely completely rule out the diagnosis or the need for diagnostic laparoscopy^92,151,155^. In the case of low probability of appendicitis, imaging may overdiagnose or detect resolving appendicitis and lead to unnecessary appendectomies and economic cost. Thus, it is the intermediate risk group that may be targeted for imaging in the first instance. Routine imaging leads to higher costs and longer duration of hospital stay, but may be justified in older people^134,156^.

Abdominal CT is generally more accurate than US in most studies, although some studies show that the effect is small if the patient’s likelihood of appendicitis before the radiological examination is taken into account^157,158^. The main disadvantage of CT is the exposure to ionizing radiation, although exposure is lower today than in the studies typically used to estimate the risk of radiation-induced malignancies^159^. Nonetheless, recent studies also have confirmed an increased risk of malignancy in patients exposed to CT for suspicion of appendicitis^160^. US and occasionally MRI can be used, especially for pregnant women and children. To minimize radiation, algorithms starting with US followed by CT in cases of unclear US diagnosis are also possible^161^. The advantages and disadvantages as well as indications and contraindications for each method are described below.

### Ultrasound

US is the first-choice examination for children and pregnant women, as it involves no exposure to ionizing radiation and does not require the use of contrast^162^. An additional advantage is that it can be used for bedside examination. US has a sensitivity and specificity for appendicitis in children of approximately 85–90% and 90–95% respectively, and for adults of approximately 80–85% and 85–90% respectively^92,155,163^. The method is known to be user-dependent. Obese patients are difficult to examine. US is also inferior to CT in diagnosing appendicitis abscess.

The US findings supporting the diagnosis are a visualized appendix (absent in 34–71%)^164,165^ with a diameter over 6 mm^166–168^ and localized tenderness over the appendix on examination. A diagnostic cut-off of a diameter greater than 6 mm is often used, but the probability of appendicitis does not reach high values until the appendix diameter measures around 8 mm^166,167,169^. Thus, the commonly used cut-off of over 6 mm is more useful for excluding than confirming appendicitis.

Appendicoliths may be seen in some cases (increasing the risk of complicated appendicitis) and there is also an increased echogenicity of an inflamed appendix. Secondary signs of appendicitis such as increased echogenicity in the surrounding fat and fluid in the pouch of Douglas, thickening of the adjoining intestinal wall, and phlegmon strengthen the diagnosis^170–172^.

### Abdominal CT

Abdominal CT with intravenous contrast has a sensitivity and specificity of approximately 95% in both children and adults^155,163,173^. The diagnostic accuracy improves by the addition of contrast^174^, especially for the diagnosis of abscesses^175^, and CT is preferable to ultrasound when an appendiceal abscess is suspected.

Oral contrast usually adds nothing to the diagnosis but delays the examination by 2 h. Low-dose screening is preferable and does not impair diagnostic accuracy or patient outcomes^176–178^. In most studies on adults, low-dose examination usually means 3 mSv. In children, the dose must be adjusted according to age and weight.

Radiological findings that support the diagnosis are^179–181^:

- enlarged appendix with a diameter >6 mm
- wall thickening >2 mm
- stranding in the fat next to the appendix
- free fluid surrounding the appendix

However, the diameter of a normal appendix can vary with substantial overlap with that of an inflamed appendix, making it unreliable on its own to diagnose appendicitis^167,169,182^. As mentioned above, the likelihood of appendicitis increases with increasing appendix diameter, but values between 6 and 8 mm do not necessarily confirm the diagnosis^169,182,183^.

It is difficult to assess whether there is a perforation or to distinguish uncomplicated from complicated appendicitis with abdominal CT alone^184,185^. The likelihood of complicated appendicitis increases if there is fluid in the lower right quadrant combined with fever >39°C and white blood cell (WBC) count >15 × 10^9^/l.

The main disadvantage of a CT scan is the exposure to ionizing radiation and iodine contrast. The lifetime risk of developing radiation-induced cancer after being exposed to a radiation dose of 7 mSv, which corresponds to a standard CT scan, has been estimated to be about 0.1–0.2%^158^, but the risk varies with age. Methodological difficulties must be taken into account in interpreting these studies. The risk for the individual is difficult to estimate but at the group level, more CT scans will result in an increased number of malignancies^186^. A recent study has confirmed an increased risk of malignancy after exposure to a CT examination for suspicion of appendicitis^160^.

The indications for an abdominal CT scan are:

- long duration of symptoms (3–5 days), where abscess is suspected
- unclear diagnosis
- when there is a suspicion of malignancy (especially older patients).

The relative contraindication for an abdominal CT scan is pregnancy. When using iodine contrast, patients with renal failure (dose adjustment may be required) and possible iodine contrast allergy should be considered.

### Magnetic resonance imaging

MRI is recommended as a radiation-free alternative to CT. It is primarily used to examine pregnant women in cases where ultrasound has been inconclusive. The method has 95% sensitivity and 92% specificity^187^. It has been proposed as an alternative to US in adolescents and children. However, it is rarely used because it is an expensive examination and availability is much lower than for US and abdominal CT. It is also more demanding on cooperation, which is difficult for many children. A fluid-filled appendix is considered diagnostic if the diameter is >7 mm.

## Diagnosis of appendicitis in pregnant women

### Appendicitis in pregnant women

Appendicitis is the most common indication for non-obstetric surgery in pregnant women, occurring in approximately 1/1000–1500 pregnancies^188,189^. The incidence of appendicitis is lower in pregnant than non-pregnant women, especially in the first and third trimesters. A clear excess risk in the first weeks after delivery suggests a transient protective effect of pregnancy^46,190^.

Pregnant women represent a difficult group to study for both ethical and practical reasons. Most published reports of appendicitis during pregnancy are based on small retrospective studies, usually without comparison with non-pregnant women. Most of the few larger registry studies lack comparative information on pregnant women without appendicitis and information on the length of pregnancy. It is therefore difficult to draw firm conclusions, and the evidence for any recommendations is weak. Moreover, the conclusions of the available studies are not directly applicable to the current conditions in Sweden. A recent Swedish study highlighted the diagnostic challenges in this group of patients, with a high proportion of negative explorations especially in the third trimester^191^.

The studies that specified length of gestation did not show any increased risk of miscarriage after appendicitis in early pregnancy compared with the background risk^192^. However, an increased risk of adverse pregnancy outcomes and especially preterm delivery has been reported in women with appendicitis during pregnancy, especially if peritonitis is present^192–195^. In pregnant women who undergo surgery for appendicitis after week 24, there is a 1.4–2.8 times increased risk of preterm delivery, or an absolute excess risk between 2% and 14%^188,196^. In appendicitis with peritonitis, there is an up to four-fold increase in the risk of preterm delivery relative to appendicitis without peritonitis^192–194^.

Appendectomy before week 24 did not affect the subsequent prognosis of the pregnancy, but appendectomy later in pregnancy is associated with an increased risk of preterm delivery within 1 week after appendectomy, and an increased incidence of babies born small for gestational age^188^.

There is no randomized study comparing active *versus* expectant or conservative management of pregnant women with suspected appendicitis. Appendectomy for suspected appendicitis in pregnancy is associated with a high rate of negative appendectomies (36%)^188,195^, reflecting diagnostic difficulties. The underlying cause may be ovarian torsion, myoma, tubo-ovarian abscess, mesenteric lymphangitis, etc. The group with negative appendectomy has the same risk of preterm delivery and fetal death as patients with uncomplicated appendicitis^195^. In cases of suspected uncomplicated appendicitis, it is important to try to minimize the proportion of negative appendectomies through, for example, imaging or, in appropriate cases, expectant management with repeat examination.

#### Symptoms and clinical findings

The classic symptoms of abdominal pain, nausea, and vomiting are common in appendicitis even in pregnant women^197–199^, and are also common in pregnant women in general, making diagnosis difficult^200^.

The uterine growth may affect the localization and distribution of pain, tenderness, and peritoneal irritation in late pregnancy. Later in pregnancy, abdominal pain may be located along the entire right side of the abdomen^197^.

Direct and indirect rebound tenderness are also common symptoms of appendicitis in pregnant women, possibly with greater upward extension in late pregnancy^201,202^.

### Diagnostics in pregnant women

**Recommendation**

1. When appendicitis is suspected in pregnant women, blood tests including leucocytes, neutrophils, CRP, and a urine sample should always be taken (⊕⊕).
2. Ultrasound is the first choice (⊕), followed by MRI if available (⊕⊕⊕).
3. Observation with repeated blood tests and ultrasound in cases of suspected appendicitis is reasonable if the woman is not otherwise unwell. This does not appear to affect maternal or fetal outcome (⊕).
4. If US is inconclusive or if US and MRI are not available or would significantly delay the diagnosis, CT may be indicated if there is a sufficiently strong suspicion of complicated appendicitis unless the general condition is so affected that there is an indication for urgent surgery (⊕⊕).
5. Diagnostic laparoscopy seems safe when it comes to preterm birth and fetal loss at least in early pregnancy (<20 weeks). However, the published evidence of the risk *versus* benefits is very limited and conflicting (⊕).

Pregnant women with a fetus of viable gestational age (over 22 + 0 weeks gestational age) with acute onset of abdominal pain should first be assessed in an obstetric ward to exclude acute obstetric conditions including threatened preterm labour/delivery, placental abruption, pre-eclampsia, etc., and then, if necessary, promptly by a consultant surgeon. Before 22 + 0 weeks of pregnancy, the patient can be primarily assessed in either a surgical or gynaecological emergency department based on symptoms. If the patient is primarily assessed in a surgical emergency department, consider a gynaecological consultation to rule out gynaecological differential diagnoses, especially extrauterine pregnancy, torsion, cyst rupture, and gynaecological infection (salpingitis, tubo-ovarian abscess). We recommend that the pregnant woman with unexplained abdominal pain be managed by specialists in the respective specialty.

Clinical examination is the basis for the assessment of suspected appendicitis even in pregnant women, although the classic symptoms of appendicitis are more non-specific in this group^200^. Pregnant women (from week 20 + 0 to 6 weeks post-partum) should be assessed according to the Obstetric Norwegian Early Warning System (ONEWS, *Table 3*)^204^, as pregnancy affects physiology, which may in turn influence the assessment^205^.

**Table 3:** **Swedish version of the Obstetric National Early Warning Signs version 2 (ONEWS-2). See Spångfors *et al.*** ^203^

	Score						
Physiological parameters	3	2	1	0	1	2	3
Respiration rate per minute	<10			10–20		21–29	≥30
Oxygen saturation (%)	≤95			≥96			
Supplied oxygen	Yes			No			
Systolic blood pressure (mm Hg)	<80	80–89		90–139	140–149	150–159	≥160
Diastolic blood pressure (mm Hg)				<90	90–99	100–109	≥110
Pulse rate* per minute	<60			60–110		111–129	≥130
Level of consciousness†				Alert			CVPU
Temperatures (°C)	≤35.0		35.1–36.0	36.1–37.9	38.0–38.9		≥39

NEWS, Norwegian Early Warning System. *If heart rate is measured, it should be used instead of pulse rate in this parameter. †Level of consciousness: A = alert, C = confusion (new or worsening confusion), V = voice (responds with eye opening, speech or movement to speech/forceful calls), P = pain (responds to pain stimulation), U = unresponsive (does not respond to speech/pain stimulation).

The diagnostic value of leucocyte count in pregnant women has been questioned because of a physiological leucocytosis during pregnancy, which is relatively moderate in most cases^206,207^. According to the few studies that have analysed their role in pregnant women with suspected appendicitis, leucocyte count and the neutrophil/lymphocyte ratio have a strong diagnostic value^199,208,209^. Therefore, the investigation of inflammatory laboratory parameters (leucocyte count, neutrophils, CRP) should always be included in the primary assessment of a pregnant patient with suspected appendicitis, together with the history and clinical examination. Diagnostic scoring systems have also shown diagnostic value in suspected appendicitis in pregnant women^210^.

If there is a clinically strong suspicion of particularly advanced appendicitis (severe inflammatory reaction and clear signs of peritoneal irritation), exploration of the abdomen should be considered, as negative imaging rarely rules out appendicitis. Observation with repeated examination can be used in cases of mild symptoms and absence of systemic inflammation. For others, diagnosis should be pursued with imaging taking into account the available modalities. The first choice is abdominal US, which has no known risks in pregnancy^211^ and is readily available in most hospitals. US for appendicitis has slightly lower sensitivity and specificity in pregnant women than in non-pregnant women because of the pregnant uterus^211^.

Non-contrast MRI also has no known risks during pregnancy and has better sensitivity than US for appendicitis in pregnant women^212^. MRI is a good first- or second-line option, depending on availability, in pregnant patients^211^.

If US is inconclusive, or if US and MRI are not available or would significantly delay the diagnosis, CT may be indicated if there is a sufficiently strong suspicion^211^, as long as the condition does not indicate the need for emergency surgery. There is clear evidence that the risks associated with a negative appendectomy during pregnancy^195,202^ are greater than pursuing the diagnosis with a CT scan.

The radiation dose in abdominal CT for appendicitis is much lower than that considered to pose a risk of fetal damage^211^. However, a dose optimization/adjustment should be made to minimize the radiation dose in all CT examinations of pregnant women.

Diagnostic laparoscopy and laparoscopic appendectomy seem safe when it comes to preterm birth and fetal loss, and preferable to open surgery at least in early pregnancy (<20 weeks)^213^. However, published evidence of the risk *versus* benefits of this approach is very limited and conflicting.

It should be considered that there is a risk of delayed diagnosis/surgery in pregnant women due to diagnostic uncertainty and hesitation about surgery in a pregnant patient. It is mainly the perforated appendicitis that carries an increased risk of preterm labour, preterm birth, and other serious complications^188,193–195^. Once the decision to operate has been made, pregnant patients should be prioritized as perforation carries increased risks to the pregnancy^193,194^.

It is also important to aim for a low rate of negative appendectomies in pregnant women, as even negative appendectomies carry an increased risk of preterm birth^195,203^. A negative appendectomy may also mask other acute conditions that still require surgery, such as ovarian torsion.

## Treatment

### Preoperative optimization

Many hospitals have local checklists for preoperative preparations that describe ID labelling, skin preparation, bed cleaning, intravenous access, blood tests, etc. Whether blood grouping and antibody screening should be taken before surgery may be judged by the responsible surgeon and anaesthetist according to the type of patient and local protocols.

In the preoperative optimization of a patient with appendicitis, particular attention should be paid to intravenous fluid therapy, as the patient may have a long history of nausea and vomiting combined with fever. There are no specific studies, but it is reasonable to assume that restoration of homeostasis, including rehydration and electrolyte correction, should be undertaken before surgery. Patients with complicated appendicitis may have hyponatraemia, which should be considered in the preoperative fluid therapy: hypotonic solutions should be avoided, and intravenous fluids with a sodium concentration of 140 mmol/l are preferred^121,123,214^.

Analgesics should be given to reduce pain and discomfort. There is no evidence that the use of opioids delays the diagnosis^215^. The choice of analgesic depends on the nature of the pain and the patient.

Preoperative preparation also includes informing the patient. Information should be based on the age and maturity of the patient and should be given in a way the patient can understand. In Sweden special websites are recommended for children and adolescents and their parents (www.mediprep.se or www.narkoswebben.se).

### Antibiotic prophylaxis and postoperative antibiotics

**Recommendation**

1. A dose of broad-spectrum antibiotics should be given as prophylaxis for appendectomy (⊕⊕⊕⊕).
2. No postoperative antibiotics should be given in surgery for uncomplicated appendicitis (⊕⊕⊕⊕).
3. Postoperative antibiotics should be given after surgery for complicated appendicitis. The definitions of gangrenous and perforated appendicitis vary widely, making studies difficult to interpret. There is probably no benefit from postoperative antibiotic treatment in gangrenous appendicitis (⊕⊕), but due to the uncertainty of the evidence, a 24-h postoperative antibiotic is recommended.
4. Postoperative antibiotics should be given after surgery for perforated appendicitis, especially if adequate source control has not been achieved. Start antibiotic treatment immediately upon suspicion and continue for 3–5 days after surgery (⊕⊕⊕⊕). Treatment can be switched to an oral option as soon as clinically feasible (⊕⊕⊕).

Antibiotics in addition to surgery for acute appendicitis are used in the form of:

- preoperative antibiotic prophylaxis
- antibiotic treatment prior to surgery in a patient with generalized peritonitis or septic condition
- postoperative antibiotic treatment in complicated appendicitis (gangrenous/perforated) to reduce the risk of wound infection and intra-abdominal abscess.

#### Antibiotic prophylaxis

Antibiotic prophylaxis during appendectomy reduces the risk of wound infection and intraabdominal abscess^216^. The antibiotic should be given 0.5–1 h before skin incision^216–218^.

The type of antibiotic prophylaxis varies in different studies and the importance of different resistance patterns should be taken into account. Different regimens have been studied in RCTs^219–223^. The recommended antibiotic prophylaxis for appendectomy in adults and children in Sweden is trimethoprim/sulfamethoxazole and metronidazole, or metronidazole alone^217,218,224^.

#### Postoperative antibiotic treatment

After surgery for uncomplicated (phlegmonous) appendicitis, no more antibiotics should be given, as they do not reduce the risk of postoperative wound infections^216,223,225^ but instead increase the risk of antibiotic-related complications^226,227^.

The importance of postoperative antibiotic treatment in complicated appendicitis appears to be largely related to the degree of intra-abdominal contamination and source control^228^. In non-perforated, complicated appendicitis (gangrenous appendicitis), the need for postoperative antibiotic treatment is controversial. A major contributing factor is the varying definition of appendicitis severity and the poor correlation between the intraoperative assessment and the subsequent histopathological examination^8,118,214^. In several well-designed studies, but with varying definitions of gangrenous appendicitis, there was no clear evidence that postoperative antibiotic treatment may reduce the risk of postoperative infection^216,222,224,226,228,229^. As the combined results of the studies are still considered uncertain and large studies are needed to demonstrate effects for such a rare outcome, and as the risk of a few additional antibiotic doses is considered very small, we still recommend a total of one day of antibiotic treatment in gangrenous appendicitis, in line with Swedish national guidelines (*Table 4*).

**Table 4:** Recommended prophylaxis and postoperative antibiotic therapy in acute appendicitis

Population	Prophylaxis*	Gangrenous	Perforated†
Adults	Trimethoprim/sulfamethoxazole (16 mg/ml + 80 mg/ml) 10 ml × 1 iv + metronidazole 1.5 iv or metronidazole alone 1.5 g iv	Piperacillin/tazobactam 4 g iv, 3 postoperative doses	Piperacillin/tazobactam 4 g × 3 iv or amoxicillin/clavulanic acid 875/125 mg × 3 iv + metronidazole 400 mg × 3 in, 3–5 days in total
Children	6 months to 5 years: trimethoprim/sulfamethoxazole (16 mg/ml + 80 mg/ml) 2.5 ml iv + metronidazole 20 mg/kg iv 6–12 years: trimethoprim/sulfamethoxazole (16 mg/ml + 80 mg/ml) 5 ml iv + metronidazole 20 mg/kg iv Over 12 years: trimethoprim/sulfamethoxazole (16 mg/ml + 80 mg/ml) 10 ml iv + metronidazole 20 mg/kg iv or metronidazole only 20 mg/kg	2–12 years: 100 mg piperacillin/12.5 mg tazobactam per kg body weight, 3 postoperative doses	2–12 years: 100 mg piperacillin/12.5 mg tazobactam per kg body weight/every 8 h
Pregnant women	Cefuroxime 1.5 g × 1 iv + metronidazole 1.5 g × 1 iv	Piperacillin/tazobactam 4 g iv, 3 postoperative doses	Piperacillin/tazobactam 4 g × 3 iv or amoxicillin/clavulanic acid 875/125 mg × 3 iv + metronidazole 400 mg × 3 in, a total of 5 days

*In patients allergic to sulfonamides: cefuroxime 1.5 g × 1 iv; †alternatives to the oral treatment are ciprofloxacin 500 mg × 2 and metronidazole 400 mg × 3, or ciprofloxacin 500 mg × 2 and clindamycin 300 mg × 3, or trimethoprim/sulfamethoxazole 160/800 mg × 2.

The single most important risk factor for the development of postoperative infections is perforation of the appendix^230^. Visible perforation also carries a significantly higher risk of infectious complications than necrosis and presence of pus in the abdomen without visible perforation^231^. Patients with perforated appendicitis should therefore always receive postoperative antibiotic treatment. However, there is no full consensus on how long this treatment should be, and the available studies are extremely heterogeneous as regards definition of perforated appendicitis, types of antibiotics used, and classification of postoperative intra-abdominal infections. Altogether, current evidence indicates that antibiotic treatment for 3–5 days is fully sufficient, and many studies showed that even shorter schedules are safe^232–237^ (*Table 4*).

Treatment can be switched to oral therapy post surgery when bowel function is restored, leucocyte count is normalized, and the patient is afebrile^3,223,238–241^. Of course, individual judgement is always required, and some patients may need a longer treatment, including a longer oral course after the intravenous course.

### Thrombosis prophylaxis

**Recommendation**

1. Thrombosis prophylaxis is not routinely recommended in surgery for acute appendicitis.
2. If the patient is over 40 years old and the operation lasts longer than 60 min, thrombosis prophylaxis should be considered.
3. Thrombosis prophylaxis should be administered to patients with clear risk factors for thrombosis, including pregnancy.

Thrombosis prophylaxis is not routinely required in laparoscopic or open surgery for acute appendicitis. However, for patients over 40 years of age with an estimated operating time longer than 60 min, it should be considered. Thrombosis prophylaxis is also indicated in cases with risk factors such as obesity, malignancy, previous thrombosis/embolism, if the patient is on oral contraceptives, and if it is expected that the patient will not be mobilized during the first postoperative days. All pregnant patients should receive thrombosis prophylaxis.

### Surgical treatment

**Recommendation**

1. In assumed complicated appendicitis (not abscess and phlegmon), patients should be optimized and operated within 6 (–8) h for the sake of well-being and to reduce the risk of complications (⊕⊕).
2. In assumed uncomplicated appendicitis, the risk of complications is not increased by up to 24 h from arrival at the emergency department to surgery (⊕⊕⊕⊕).
3. Laparoscopic appendectomy is recommended as the first choice for all patient groups and for both uncomplicated and complicated appendicitis (not abscess and phlegmon) (⊕⊕⊕⊕).
4. The standard three-port technique for laparoscopic appendectomy is recommended for both adults and children (⊕⊕).
5. Open appendectomy may need to be used in late pregnancy and in case of contraindication to laparoscopy, but it is always a matter of judgement in each case.
6. In complicated appendicitis with free fluid in the abdomen, aspiration of the fluid is recommended but not peritoneal lavage (⊕⊕⊕).
7. Postoperative abdominal drain is not recommended (⊕⊕⊕⊕).

#### Prioritization of operations

If the condition indicates uncomplicated appendicitis and if the inflammatory reaction is mild or moderate, several studies have shown that surgery can be delayed for up to 24 h without risk of perforation or other complications such as postoperative wound infection, intra-abdominal abscess, small bowel ileus, need for reoperation or readmission^73–75,79,242–245^. This means that in this situation an operation can be postponed until daytime or the next day without risk (*Table 5*). However, the patient should be monitored and the operation be brought forward if the general condition worsens or signs of peritonitis develop. The waiting time should also be minimized for the sake of well-being. A decision to operate may be changed if there is clinical improvement with decreasing inflammatory markers or temperature during the waiting period, which may indicate spontaneous resolution^73–75^. Like others, pregnant women with uncomplicated appendicitis have no increased risk for progression of the disease if operated on within 24 h.

**Table 5:** Prioritization of operations

Operation prioritization	Emergency priority 2 h	Emergency priority 6–8 h	Emergency priority under 24 h
Severity level	Sepsis	Complicated appendicitis (not abscess or phlegmon)	Uncomplicated appendicitis

If the appendix looks normal at laparoscopy and no other pathology explaining the symptoms is found, the management is not clear, but appendectomy can be recommended, especially in the elderly^246^.

In patients with signs of generalized peritonitis and possible sepsis, suggesting complicated appendicitis, antibiotic treatment should be initiated immediately, and the patient be optimized in terms of rehydration and correction of electrolytes. This optimization may take up to 6–8 h. Thrombosis prophylaxis should be used if necessary (see above). After optimization, surgical treatment should be undertaken without delay.

However, if the clinical assessment suggests complicated appendicitis with signs of an abscess or phlegmon, conservative treatment with antibiotics should be used in the first instance. For abscesses of 3–5 cm in diameter, percutaneous drainage should also be considered. Upon exploration and discovery of a large inflammatory mass (phlegmon) without free peritonitis, discontinuing the surgery and continuing conservative treatment with antibiotics should be considered as further dissection and attempted appendectomy is associated with the risk of colon resection (see Primary treatment with antibiotics).

#### Surgical techniques

Laparoscopic appendectomy is recommended as the first choice for all patients except in late pregnancy or when there is a contraindication to laparoscopy. In meta-analyses and larger studies laparoscopic appendectomy demonstrated several advantages over open appendectomy in terms of pain, wound infection, time to food intake, length of stay, recovery, cost, and quality of life. These findings remained true in various patient subgroups including children, adults, the elderly, and the obese^247–250^. However, many of these studies display substantial selection bias as complicated appendicitis is more commonly treated by open appendectomy.

To summarize, an appendectomy can be performed with good results by any method. Open surgery is recommended for pregnant women in the third trimester or if laparoscopy fails. It can also be considered if the patient is known to have adhesions from previous surgery. Unlike open surgery, laparoscopy provides an excellent diagnostic opportunity.

Single-port laparoscopic appendectomy has been compared with ‘conventional’ three-port laparoscopic appendectomy in several meta-analyses of randomized trials^251–256^. In summary, the conventional three-port laparoscopic appendectomy is recommended as it is significantly more accessible from an instrument and expertise point of view and in adults results in less pain and has a shorter operating time.

Dissection of the mesoappendix can be done with monopolar electrocoagulation, bipolar energy, metal clips, stapler, endoloops, Ligasure, or Harmonic Scalpel at the operator’s preference as no major clinical advantages have been found for any of them^257,258^. Dissection of the mesoappendix with diathermy is most common, but a stapler can be used if the mesoappendix is clearly oedematous. The choice of the dissection instrument can be determined by surgeon expertise and hospital resources. Division of the appendix in adults or children, regardless of uncomplicated or complicated appendicitis, is recommended to be performed with ligature, endoloop, metal clips, or stapler. In case of an inflamed caecum or large appendix diameter, stapler should be preferred. Most available studies show no differences between stapler and endoloop closure^259,260^. A recent Cochrane review did not show any major differences between the different methods of appendix closure in uncomplicated appendicitis^261^. Several studies reported that clips are inexpensive and provide similar results as other more expensive alternatives, thus representing a cost-effective option for appendix ligation in uncomplicated appendicitis. Yet, most of them did not adjust for the severity of the appendicitis, limiting the strength of their conclusions. Logically, staplers should be preferred if the appendix base is thick and the tissue is severely inflamed^259–266^.

Regarding stump management, invagination is not recommended. In fact, a systematic review of several RCTs showed shorter operation time, lower incidence of ileus, and faster recovery after simple ligation^266^. Additionally, an invaginated stump might be mistaken for a polyp in a future radiological examination of the colon. It is important that the length of the stump should not exceed 0.5 cm, as the risk of stump appendicitis has been described from this length onwards^267^.

In perforated appendicitis, aspiration with lavage offers no advantage over aspiration without irrigation in either children or adults. Lavage does not reduce the risk of intra-abdominal infection, wound infection, or length of stay; instead, it likely only increases operative time^268,269^.

Postoperative abdominal drainage after appendectomy for complicated appendicitis has not been associated with a reduction in abscesses or wound infections, athough it results in longer hospital stay, and, in children, is often linked to higher 30-day morbidity rates^270–272^.

#### Surgical treatment in pregnant women

Both the laparoscopic and open approaches are viable options for appendectomy in pregnant women, although reports on laparoscopic appendectomies during the third trimester (28 weeks of gestation or more) are scarce. This is because the abdomen is increasingly occupied by the uterus as the pregnancy progresses, so open procedures are often preferred.

Laparoscopic appendectomy has not been associated with an increased risk of birth defects or developmental delay, either compared to open surgery or to healthy pregnant women who did not undergo surgery during pregnancy^273,274^. When it comes to fetal outcome, randomized studies are lacking and the observational studies available are of low quality with a high risk of bias^275–277^. With regard to fetal death (miscarriage and intrauterine death) and preterm birth, there is no randomized study comparing open and laparoscopic appendectomy, but a meta-analysis of observational studies reported an increased risk of miscarriage/fetal death after laparoscopic appendectomy (5.3% compared to 3.0% after open surgery)^275^. Yet, literature limitations are substantial. For example, a registry study with a large impact on several meta-analyses due to its large sample size has a clear weakness in that it does not distinguish between miscarriages and fetal death in viable gestation^195,275^. This results in serious bias. In fact, although miscarriage is common in the first trimester, fetal death in later gestation is a rare event. However, the laparoscopic approach represents the preferred option in early pregnancy, whereas open surgery is more common in later pregnancy^278^.

An individual assessment of the pregnant woman by the surgeon performing the appendectomy must always be made and the final choice of surgical technique should be based on experience, competence, and personal judgement. For example, the size of the uterus can vary among different patients in the same week of pregnancy, and multiple pregnancy is associated with larger size. In pregnancies from 22 + 0 weeks, obstetricians should be consulted in the preoperative planning to assess the need for perioperative fetal monitoring or prophylactic tocolysis.

If a laparoscopic appendectomy is technically feasible, it involves less surgical trauma than open abdominal surgery and allows better visualization of the abdomen^279^. Notably, the position of the appendix base does not appear to change with uterine growth^280,281^.

Compared with the open approach, laparoscopic appendectomy in pregnant women has been associated with shorter operative time, shorter hospital stay, and reduced risk of wound infection^282,283^. However, the available studies are not randomized and there was a higher proportion of advanced appendicitis in the open surgery group, possibly biasing the results^284^.

Recommendations for appendectomy in pregnant women include the following:

- Position a wedge cushion on the right side of the patient to tilt the uterus to the left, minimizing pressure on the inferior vena cava (this does not apply in the first trimester of pregnancy).
- In laparoscopy, perform open entry to minimize the risk of damaging the uterus during insufflation and insertion of the first trocar.
- Adjust the position of the trocars according to the size of the uterus.
- Limit the intra-abdominal pressure to a maximum of 15 mmHg^276^.

## Primary treatment with antibiotics

### Primary treatment of abscess or phlegmon with antibiotics

**Recommendation**

1. Patients with appendicitis abscess should be treated primarily with intravenous antibiotics and drainage. Phlegmon should be treated primarily with intravenous antibiotics (⊕⊕⊕⊕).
2. Planned interval appendectomy after conservatively treated abscess or phlegmon is not routinely recommended except in cases where malignancy is suspected and/or unclear findings at follow-up remain (⊕⊕).
3. Patients who have been treated conservatively for appendicitis abscess or phlegmon should be followed up with CT or colonoscopy (sometimes a combination of both) as there may be an underlying malignancy. This applies especially for patients aged over 40 years.

In patients with symptoms lasting more than 3 days, imaging should always be performed as the likelihood of an abscess or phlegmon is increased. Three-digit CRP values also increase the risk of abscess or phlegmon. In these patients, physical examination may reveal a tender palpable mass in the right iliac fossa. Imaging often confirms signs of a localized abscess or a large inflammatory infiltrate (phlegmon).

Acute surgical treatment of appendiceal abscess with laparoscopy is possible but not recommended as it is associated with significant morbidity rates in most studies^3,285–287^. Conservative treatment is associated with a significantly lower risk of wound infection, abscess, bowel obstruction, and need of reoperation^288–290^. Dissection can be difficult, often leading to conversion to open surgery (10%), increased risk of resection of the right colon (10%), and an incomplete resection of the appendix (13%)^285^.

Larger studies report that about 25% of cases treated with intravenous antibiotics and drains fail and require surgery^225,291^. Many authors recommend elective appendectomy after successful conservative treatment of appendicitis to prevent recurrence. This approach is, however, questionable as the recurrence risk is approximatively 10–15% in the first year, so 85% of the patients would be operated on unnecessarily. In other words, six patients need to be treated to prevent recurrence in one patient. Moreover, planned appendectomy has been associated with higher morbidity rates compared to wait-and-see^292^. For these reasons, planned appendectomy after successful conservative treatment is not recommended. Patients should rather be advised to seek medical care promptly if their symptoms recur.

Patients with an abscess or phlegmon should thus be treated primarily with intravenous broad-spectrum antibiotics, providing coverage against Gram-negative bacteria and anaerobic organisms^293,294^. Treatment should last at least 5 days. For abscesses from 3 to 5 cm in diameter, percutaneous drainage may be appropriate, whereas abscesses over 5 cm should always be drained^295,296^. If aspiration or drainage fails to yield pus but returns mucus, it may be a mucinous tumour in the appendix or caecum, an appendicular mucocele.

If a large inflammatory infiltrate is unexpectedly found during surgery, the procedure should be halted, and conservative treatment should be initiated. If the perioperative assessment indicates that the appendix must be removed and there is a suspicion of malignancy without signs of abdominal spread, a right-sided hemicolectomy is recommended, provided it can be performed in a macroscopically radical manner with central ligation of the vessels. If there is uncertainty as to whether the patient should undergo peritonectomy with hyperthermic intra-peritoneal chemotherapy (HIPEC), contact with a specialist centre is advised in the first instance, and in the second instance an ileocaecal resection should be performed to obtain a diagnosis. Biopsy for histopathological examination (or frozen section) may be indicated. Avoid performing a non-radical operation, as this worsens the patient’s prognosis.

Patients who have been treated conservatively for appendicitis abscess should be followed up with CT colonography or colonoscopy as there may be an underlying malignancy. This applies particularly to patients over 40 years of age, or those with a history suspicious for malignancy^297,298^.

#### Primary treatment with antibiotics of abscess or phlegmon in pregnant women

There is limited evidence for primary antibiotic treatment of appendicitis abscess or phlegmon in pregnant women^299,300^. The risks of preterm labour and preterm birth should be considered when deciding on clinical management. Management should derive from multidisciplinary discussion in each of these few cases.

In a pregnant woman with a confirmed primary appendicitis abscess, the risk of the infection triggering premature contractions supports surgical treatment. A recently published registry study of over 8000 women with complicated appendicitis favours prompt surgery over conservative treatment. Even if surgery was successful, the risk of chorioamnionitis increased. If conservative treatment failed, the risk of premature labour, delivery, and fetal death also increased^301^.

The problem is illustrated by a case report of two pregnant women treated conservatively for an appendicitis abscess with only intravenous antibiotics and no drainage of the abscess^302^. In one case, the woman recovered and had an uncomplicated delivery at full term. In the second case, the woman made a temporary recovery but recurred 5 weeks later with acute appendicitis. A new attempt at conservative treatment with antibiotics was made. This time the woman developed preterm labour and underwent an emergency caesarean section at 34 weeks’ gestation due to breech presentation and threat of preterm birth, and an appendectomy was performed at the same time.

### Primary treatment of uncomplicated appendicitis with antibiotics

**Recommendation**

1. In two randomized trials, antibiotic treatment was not significantly better than placebo and therefore cannot be recommended for the treatment of uncomplicated appendicitis (⊕⊕⊕⊕).

Several studies have randomized patients with uncomplicated appendicitis to either treatment with antibiotics or surgery, not least from Sweden^303–305^. One relevant issue with these trials is patient selection, as no method can distinguish uncomplicated from complicated appendicitis or non-inflamed appendix with certainty^306^. Scoring systems combining clinical and radiological factors have been proposed, but prospective evaluations in patients with suspected appendicitis are lacking^307^.

In most published series, antibiotic treatment is initiated after perforated appendicitis has been ruled out. Usually the patient is hospitalized, fasting and prepared for possible surgery in case of treatment failure. Upon improvement, enteral administration is started as soon as tolerated, and oral antibiotics are given with a total treatment duration of 7–10 days. Antibiotic treatment involves the use of high-dose broad-spectrum antibiotics, which are associated with common short- and long-term adverse effects^308,309^.

Treatment results indicate a moderate risk of initial treatment failure (approximately 10%) and a risk of appendicitis recurrence/appendectomy in the first year of 20–25%^310–313^. This strategy is also associated with risk of abdominal exploration for abdominal pain with negative appendectomy during follow-up^305^. Long-term data indicate that the risk of recurrence is around 40% for adults and around 20% for children^312,314^. A recent meta-analysis of eight randomized trials shows a six-fold increased risk of readmission in the antibiotic group relative to the surgery group within 1 year from the appendicitis episode^315^.

Many studies suggest that spontaneous resolving of appendicitis is common^22,59,61–68,316,317^. The true therapeutic effect of antibiotics has so far been investigated in only two placebo-controlled trials^65,66^. Park *et al*. showed 97% primary resolution regardless of whether patients received antibiotics or saline^65^. In the APPAC III study, no significant difference could be detected between antibiotic treatment (97%) and placebo (87%)^66^. In patients with uncomplicated appendicitis diagnosed by ultrasound, a majority healed on supportive care alone^67^. This suggests that a large proportion of uncomplicated appendicitis can heal without surgery, with or without the support of antibiotic therapy.

Ongoing studies aim to further characterize which patients may benefit from non-surgical treatment and whether the appendicitis can heal with or without antibiotics in these patients. Future studies should optimally consist of randomization to three arms (placebo, antibiotics, surgery).

There is currently no evidence that successful non-operative antibiotic treatment for uncomplicated appendicitis is associated with an increased risk of developing malignant disease that could have been detected at the time of appendicitis. Primary antibiotic treatment of uncomplicated appendicitis in pregnant women is not recommended.

## Nursing care

**Recommendation**

1. The enhanced recovery after surgery (ERAS) concept speeds up recovery, shortens hospitalization time, and seems to lead to fewer reoperations (⊕⊕⊕).
2. Children who have undergone laparoscopic appendectomy for uncomplicated appendicitis can be discharged from hospital on the same day (⊕⊕).
3. Urinary catheters and central access are not indicated in children with complicated appendicitis (⊕⊕).
4. Pain relief for children at home usually consists of paracetamol and non-steroidal anti-inflammatory drugs (NSAIDs).

### ERAS

The ERAS concept has been studied in both children and adults undergoing surgery for acute appendicitis in relatively small but often well-designed studies, and appears to result in faster recovery, shorter length of hospital stay, and fewer reoperations^318–327^. Many studies focus on same-day discharge after laparoscopic appendectomy for uncomplicated appendicitis, but other studies also highlight the role of ERAS in the postoperative care of complicated appendicitis. It should be emphasized that ERAS is not only about nursing but is a comprehensive model involving all professions in the perioperative care process.

Each hospital and clinic should develop its own procedures, but the focus should be on:

- Preoperative resuscitation with restoration of fluid balance, analgesia and information on postoperative care before surgery.
- Avoidance of drains, central venous catheters, and urinary catheters if possible.
- Pain management with non-opioids.
- Rapid mobilization.
- Starting antiemetics immediately and oral intake of food and drink as soon as possible post surgery.
- Usage of as short a course of antibiotics as possible.
- 7–14 days’ sick leave is often sufficient.

### Children

The postoperative care of children differs from that of adults. Specialized expertise in the care of children and adolescents is always preferable. It is important to establish a good relationship with the child at an early stage and to continuously inform them about what will happen in the hospital at a level appropriate to their maturity. Children who are hospitalized should have checks using evidence-based scales (for example Pediatric Early Warning Scores, or PEWS).

#### Circulation

Children with appendicitis may have an elevated temperature, which should be monitored preferably every 4–6 h or before administration of antipyretics. Postoperative fever may indicate complications such as wound infection, urinary tract infection, pneumonia, or most commonly intra-abdominal abscess. Children with suspected or confirmed appendicitis usually receive intravenous fluid therapy. There appears to be no benefit for the perioperative use of central venous catheter, even in complicated appendicitis^328^.

#### Elimination

It is important to check that the child urinates both before and after surgery. Bladder volume in children can vary, but an approximate size can be calculated using the general formula: ‘child’s age in years × 30 + 30 ml’. There is no evidence to suggest that children with perforated appendicitis, either with or without postoperative opioid therapy, have a particularly high risk of postoperative urinary retention^329,330^. However, micturition should be monitored during the first 24 h post surgery. Urinary retention may also be a sign of postoperative constipation, pain, or an abscess in the small pelvis. Midazolam, sometimes given before procedures, also increases the risk of urinary retention. The child’s normal bowel habits may also be affected by the appendicitis, the surgery and anaesthesia, and post-operative antibiotics. Changes in bowel habits can lead to abdominal pain.

#### Nutrition

Children with appendicitis often present with reduced appetite and sometimes vomiting. Surgery also involves preoperative fasting. Children who start eating early after surgery and have normal bowel movements can be discharged from hospital earlier than children who have been fasting longer post surgery. Children who start eating early will also experience fewer feelings of hunger, thirst, and pain. Parenteral nutrition does not seem to reduce complications in postoperative care after complicated appendicitis^331^. Wait for the child’s signal and start offering a drink or ice cream. Once this has gone well, you can move on to food.

#### Pain

Pain associated with appendicitis is common and pain relief is provided as needed. To estimate pain and evaluate the effect of analgesics, it is appropriate to use a validated pain rating scale. Which scale to use depends, among other things, on the age and maturity of the child, and the scale implemented at the workplace. Pain can be aggravated by anxiety and specific instruments have been developed and validated to measure anxiety as well. The most commonly used pain scales in children are the Faces Pain Scale—Revised (FPS-R) (4–16 years), FLACC (Face, Legs, Activity, Cry, and Consolability) scale, for procedure-related pain, and Astrid Lindgren Children’s Hospital Pain Scale (ALPS II) (0–3 years). From school age, the Visual Analogue Scale (VAS) can also be used. Most children can be relieved with paracetamol and NSAIDs after an appendectomy. If repeated opioid pain relief is needed post surgery, a surgical complication should be suspected.

#### Activity

Early postoperative mobilization is helpful to prevent abdominal pain caused by gas in the bowel. From the perspective of patient safety and parental satisfaction, children who have undergone laparoscopic appendectomy for uncomplicated appendicitis can be discharged from hospital on the same day, provided their general condition permits^330–334^.

#### Skin

Postoperative wound infections can be prevented by a single dose of antibiotics, but the surgical wound(s) should always be inspected.

#### Social and information

Children in hospital have the right to have parents or other carers with them throughout their stay. Children and parents should receive information about illness, treatment, and care in an understandable way (Nordic Association for the Needs of Sick Children, NOBAB).

### Pregnant women

Pregnant women with acute appendicitis after week 21 should be admitted to a hospital with adequate obstetric and neonatal expertise to assess, deliver, and care for the baby, should the need arise. The care of pregnant women should always be done in consultation with an experienced obstetrician.

If there is a risk of preterm labour after 22 + 0 weeks of gestation (viable gestational age), the patient should be monitored for signs of preterm labour associated with acute appendicitis. In consultation with an obstetrician, consideration should be given to admit the patient to a maternity ward so that labour can be identified and anti-labour drugs or cortisone for lung maturation can be started in time if needed. Patients at 22–28 weeks of gestation with a strong suspicion of appendicitis should preferably be treated at one of the regional centres with expertise in preterm care.

## Follow-up after treatment of appendicitis

**Recommendation**

1. PAD should be made routinely in all patients (⊕⊕). If the resources are scarce, all appendices in patients >40 years of age should undergo PAD to avoid missing an alternative diagnosis. To evaluate the quality of the clinic, all consecutive appendectomy specimens should be analysed at least for a period of time
2. Follow-up is not necessary if PAD shows acute appendicitis.

### Objectives of the follow-up

There are several reasons for following-up some of the patients who are diagnosed with and treated and cared for appendicitis. This includes to detect postoperative complications, follow-up after conservative management and for abnormal PAD. In addition, patients can be followed up within the framework of research and quality improvement.

### Follow-up after appendectomy

#### Postoperative complications

Possible surgical complications include wound infection (1.2–12%), abscess^22^, small bowel ileus (0–1.9%), wound rupture, stump leakage, and stump appendicitis, which can occur if the stump is over 0.5 cm^267^.

Follow-up and further treatment after appendectomy is entirely determined by PAD. In phlegmonous, gangrenous, and/or perforated appendicitis without other pathology, follow-up is not necessary. It is important to send all appendiceal specimens for pathological analysis as approximately 2% of removed appendixes in adult patients may contain other pathologies requiring treatment, which would otherwise go undetected^333,334^. PAD is also important for research and quality assurance purposes. We therefore recommend consistently sending all appendix preparations for PAD, also in children. For histopathological diagnostic criteria for appendicitis, please refer to the following document: Appendix, Swedish Society of Pathology (KVAST)^335^.

Neuroendocrine tumours (NET) are the most common tumour detected on PAD (0.13–2.4%). Other unexpected findings may include diverticulitis (1.2%), tuberculosis (0.08%), endometriosis (3.6%), adenocarcinoma (<1%), and mucinous cystadenoma (0.2–0.6%)^336^. Depending on the diagnosis, further management varies from no further treatment to right-sided hemicolectomy or to peritonectomy with HIPEC^336^.

Finally, it should be noted that patients undergoing negative appendectomy appear to have increased mortality rates in both the short and long term in major epidemiological studies^25^. The cause can probably be partly explained by other underlying undetected pathology or co-morbidities.

### Follow-up after spontaneously resolved appendicitis

Patients should be informed that recurrence might occur and that they should seek emergency treatment if symptoms reappear. No active follow-up is otherwise required.

### Follow-up after conservative treatment of appendicitis abscess or phlegmon

**Recommendation**

1. Children do not need follow-up.
2. People under 40 years of age do not require follow-up unless their medical history suggests a malignant genesis.
3. People over 40 years of age are recommended to have a CT colonography or colonoscopy after 6–8 weeks to rule out malignancy (⊕). The findings on the abdominal CT scan can guide the selection of the most appropriate test.
4. Elective surgery after non-operative successful treatment is not recommended with the following exceptions (⊕⊕):
  ○ the patient has recurrent appendicitis symptoms (relapse)
  ○ malignancy is suspected at follow-up.

Follow-up of complicated appendicitis with abscess or phlegmon that has been successfully treated with antibiotics with or without percutaneous drainage is done primarily to rule out malignancy, and secondarily to detect other disease in the area, such as inflammatory bowel disease. Malignancy in the appendix is uncommon, with an incidence of about 1% of all appendectomies, about 50% of which are carcinoids. The proportion of adenocarcinoma is 0.08–0.2% of all appendectomies according to older studies.

The risk of a malignancy increases with age^337–339^. In one study the incidence of appendix malignancy in men was twice as high among those over 60 years old compared to those under age 60. Numerous studies have investigated malignancy risk in the follow-up of non-surgically treated primary complicated appendicitis (abscess or phlegmon)^292,293,297,340–342^, and reported malignancy in 3–17% of the patients over 40 years of age.

When needed, the type of follow-up examination should be chosen based on the location of the pathology identified on the abdominal CT scan. For example, wall thickening in the caecum should prompt a colonoscopy in the first instance, whereas pathology at the tip of the appendix is best investigated with CT colonography.

When it comes to elective appendectomy after the acute stage, studies in both young and old patients show that the benefit, in terms of preventing recurrence and missed diagnoses, is small to society, but represents a major effort for the individual patient with a dubious benefit in most cases. Instead, depending on age and risk factors for other diseases, follow-up after 6–8 weeks is preferred^291–293,297,342,343^.

### Long-term outcomes following surgery for acute appendicitis

Abdominal wall hernia and adhesion ileus are the main long-term complications of appendectomy. The risk of adhesive small bowel obstruction is about 1.5% after 15 years of follow-up for both open and laparoscopic appendectomy^344^. The risk for children appears to be about the same for complicated appendicitis, whereas uncomplicated appendicitis has a lower risk of about 0.25%. Severe intraperitoneal inflammation and perforated appendicitis are independent predictors of future adhesive ileus^345^. Conversion to open surgery is more common in perforated appendicitis, which causes a selection bias in many studies comparing open and laparoscopic appendectomy. Many studies have not adjusted for this bias, so the risk of laparoscopic surgery compared to open surgery is often underestimated. Furthermore, most studies and the largest meta-analyses of children and adults show no difference between laparoscopic and open appendectomy^41,248–250,346–348^, especially when only randomized studies are included^249^. In conclusion, it is the degree of inflammation and not the surgical method that seems to be the main determinant of the risk of adhesive ileus after appendectomy.

The risk of abdominal wall hernia after open appendectomy has been reported to be 0.7% after a median follow-up of 6.5 years in four studies^349^. The risk of abdominal wall hernia after laparoscopic appendectomy is poorly studied. For all types of laparoscopic surgery, it is reported to be 0.1% at 22 months of follow-up^350^.

An association with increased or decreased risk of several conditions after appendectomy has been reported^351,352^. In most cases they can be explained by selection. In some cases, common underlying pathogenetic mechanisms between appendicitis and the other disease are likely to be present. This applies to the observed lower risk of developing ulcerative colitis in patients that had appendectomy for appendicitis before age 20 whereas no such effect is seen after negative appendectomy or appendectomy for other reasons or appendectomy for appendicitis after age 20, suggesting an inverse pathogenetic relationship between appendicitis in young patients and ulcerative colitis^43,44,353^. At present, there is no firm evidence of a causal relationship between appendectomy and future increased or decreased risk of any condition. No impact on female fertility has been observed^354^.

### Follow-up responsibilities

PAD results should be followed up by the operating surgeon. All surgical patients should receive a written response with the results of the PAD except in cases where malignancy is found, which require personal contact. The discharging physician on the ward is most responsible for ensuring that the patient receives their planned follow-up. Any follow-up CT colonography or colonoscopy is ordered at discharge according to standard procedures. If primary care is to order the examination, a clear referral must be issued. Any sick leave letter and prescription have to be provided by the discharging doctor.

## Quality registers and quality indicators

There is no quality register for acute appendicitis in Sweden. The group behind these guidelines believes that it is reasonable to discuss the introduction of such a register, given the likely variation in management between different regions and hospitals and the commonality of the condition. A quality register could relatively easily map the differences in management that we believe exist based on the differences in incidence between regions and the answers to the questionnaire that we sent out at the beginning of the group’s work. *Table 6* displays a list of the potential quality indicators that were identified during the development of the guidelines and through a systematic literature review. This process is described in detail in the Supplementary material. Valuable information from patient interviews is also included.

**Table 6:** **Quality indicators and target levels, based on the group’s experience and a systematic review of the literature (see**  *Supplementary material*  **for details)**

Variable	Source	Area	Problem with measurement method	Target levels
Blood tests taken (WBC, CRP, neutrophils, sodium)	Missing	Investigation		100%
Time of arrival—pain relief	Missing	PROM	Arrival is difficult to register. The degree of pain also matters	
Risk-stratification by scoring system	Missing	Investigation		100%
Proportion of ultrasound	Missing	Investigation	Needs to be related to the patient’s age, condition, and risk of appendicitis according to scoring system	
Rate of abdominal CT scan	Missing	Investigation	Needs to be related to the patient’s age, condition and risk of appendicitis according to scoring system	<10% in uncomplicated appendicitis in younger patients
Number of appendectomies	PAR	Treatment	Need population-based data for the health unit	
Antibiotic prophylaxis and type	Missing	Treatment		100%
Method of operation: laparoscopy, open, conversion	PAR SPOR	Treatment	Needs to be partially related to the patient’s condition and any previous interventions	>90% should be operated laparoscopically
Drainage treatment for abscesses	Missing	Treatment	Depends on the size and location of the abscess and the patient’s condition	>90% should receive drainage of abscesses > 4 cm
Negative appendectomy/exploration	PAR	Treatment	Need PAD for estimation	<5%
PAD sent	Missing	Treatment	Needed for identifying negative appendectomies	100%
Wound infection	PAR	Postoperative care	Should be verified by cultivation. Difficult to register	<5%
Wound rupture	PAR	Postoperative care		<1%
Reoperation, including interventional radiology procedures	PAR SPOR	Postoperative care		<5%
Emergency department return visit < 30 days	PAR	Postoperative care	Depends on type of appendicitis and intervention	<5%
Readmission < 30 days	PAR	Postoperative care	Depends on type of appendicitis and intervention	<5%
Length of hospital stay	PAR SPOR	Postoperative care	Depends on type of appendicitis and intervention	
Time to return to school or work	Missing	PROM	Depends on type of appendicitis and intervention	
Mortality	PAR	Postoperative care		0%

Missing source means that there is currently no register available for linkage. PAR, National Board of Health and Welfare’s patient register; SPOR, Swedish perioperative register; PROM, Patient Reported Outcome Measures.

### Available registers

Demographic and socioeconomic variables such as age, gender, and country of birth can be obtained from the National Patient Register, as well as diagnosis codes, procedure codes, level and duration of care, type of hospital, readmission, and certain short- and long-term complications and co-morbidity. A major disadvantage at the moment is that the ICD-10 codes for appendicitis make it impossible to classify the severity of appendicitis correctly. It is not possible to distinguish uncomplicated appendicitis, perforated appendicitis, and appendicitis abscess, let alone distinguish between phlegmonous and gangrenous appendicitis. In the latest version of the ICD-10, the physician must decide whether there is peritonitis, which is a very subjective judgement. We hope that the next version can classify the appendicitis into non-perforated appendicitis (preferably with further division into phlegmonous and gangrenous), perforated appendicitis, appendicitis abscess, and phlegmon.

Additional data, especially perioperative data, such as surgery time, time to surgery, ASA class, weight, operator, procedure and anaesthetic codes, pain, nausea, and direct postoperative complications, could be obtained from SPOR (Swedish perioperative register). SPOR can also be used to compile outcome measures for the organizations themselves.

For studies of appendicitis/appendectomy in pregnancy, data can be obtained by cross-referencing with the Medical Birth Register (MFR, ‘Medicinska Födelse Registret’) and the Pregnancy Register (GR, ‘Graviditets Registret’).

Many data are stored in the hospital’s clinical, radiological, and laboratory departments’ digital databases, but in most cases there is no routine for linking them.

### Quality indicators and target levels

Quality indicators should primarily reflect the quality of care (knowledge-based, effective, safe, patient-centred, equitable health care and treatment in reasonable time) but also the goal of the guidelines. As an example, the variable ‘preoperative blood tests’ does not refer to the level of the test results, but whether the tests were conducted. As mentioned above, the current classification of appendicitis in the ICD-10 is controversial, and there is no national quality register for appendicitis in Sweden. This means that many of the indicators in *Table 6* cannot currently be measured reliably or at all (development indicators). Furthermore, there are currently no clear target levels for benchmarking, which is partly explained by different definitions of appendicitis in different studies. The target levels are therefore estimates based on figures from larger compilations. For some, there are no target values.

## Discussion

These Swedish guidelines for appendicitis focus on describing the natural history of the disease and the use of structured management based on risk stratification to initially assess the patient with suspected appendicitis (*Fig. 1*). Based on strong evidence as well as suitability in the Swedish context, the AIR score is recommended. This instrument weighs together the clinical and inflammatory variables that have an independent diagnostic value (vomiting, tenderness in the right iliac fossa, muscle defence, abdominal rebound tenderness, body temperature, leucocytes, proportion of neutrophils, and CRP), and has also been extensively validated internationally.

Based on this risk assessment, a decision is made regarding whether the patient with suspected appendicitis should be observed at home, taken directly to laparoscopy, or managed by repeat examination after a period of in-patient observation or further radiological investigation. The active observation with repeated clinical assessment is very valuable to identify which patients need surgical treatment. Special consideration should be given to older people, where there are more differential diagnoses, and to children and pregnant women, where diagnosis may be more difficult, and the consequences of delayed treatment may be worse.

We understand that guidelines can never serve as a perfect manual, and some cases of acute appendicitis may require different management than what is stated here. However, we strongly believe that a national guideline on appendicitis is necessary to address the wide regional variability in the management of these patients in our country. We hope that these guidelines will condense and highlight the most current knowledge about the management of appendicitis, leading to more evidence-based, uniform, and effective care throughout the country.

## Supplementary Material

zrae165_Supplementary_Data

## Data Availability

This work is a review of the literature, based on the references provided. No new data were generated.
