# Supplementary material for: Swedish national guidelines for diagnosis and management of acute appendicitis in adults and children
Source: BJS Open. 2025 Apr 9;9(2):zrae165. doi: 10.1093/bjsopen/zrae165 (PMC11980984; doi:10.1093/bjsopen/zrae165)
Supplement: zrae165_Supplementary_Data [file zrae165_supplementary_data.docx]

# Swedish national guidelines for diagnosis and management of acute appendicitis in adults and children

Martin Salö, MD, Associate Professor^1^; Catarina Tiselius, MD, Associate professor^2^;

Anders Rosemar, MD, PhD^3^; Elin Öst, RN, PhD^4^; Sara Sohlberg, MD, PhD^5^; Roland E Andersson, MD, PhD, Professor^6^1. Department of Clinical Sciences, Pediatrics, Lund University, Lund, Sweden; Department of Pediatric Surgery, Skåne University Hospital, Lund, Sweden.
2. Department of Surgery, Västmanland Hospital Västerås, Västerås, Sweden; Centre for Clinical Research, Uppsala University, Västerås, Sweden.
3. Department of Surgery, Region Västra Götaland, Sahlgrenska University Hospital Östra, Gothenburg, Sweden; Department of Surgery, Institute of Clinical Sciences, Sahlgrenska Academy, University of Gothenburg, Gothenburg, Sweden
4. Department of Pediatric Surgery, Karolinska University Hospital, Stockholm, Sweden; Department of Women's and Children's Health, Karolinska Institutet, Stockholm, Sweden
5. Department of Women´s and Children´s Health, Uppsala University, Uppsala, Sweden
6. Department of Biomedical and Clinical Sciences, Linköping University, Linköping, Sweden; Futurum Academy for Health and Care, Jönköping County Council, Jönköping, Sweden.

**Corresponding author**: Andersson RE, Futurum Läkarprogrammet hus D2, Länssjukhuset Ryhov, SE-551 85 Jönköping, **ORCID ID**: 0000-0002-1460-0248

## Supplementary Materials - Index

### Supplement search strategy for quality indicators

### Supplement References

| Supplement Search strategy We did a systematic review of the literature on quality indicators related to appendicitis in Medline, Cochrane, Cinahl and the Embase databases. The search identified a total of 27 hits (Medline n=9, Cochrane n=1, Embase n=14 Cinahl n=3), whereof 12 duplicates leaving 15 unique hits. Se all abstracts below.  **Database:** Ovid MEDLINE(R) ALL 1946 to February 09, 2021 **Date:** 2021-02-10 **No of results:** 9 ref   \| **#** \| **Searches** \| **Results** \| \| --- \| --- \| --- \| \| 1 \| exp Appendicitis/ \| 19381 \| \| 2 \| appendicitis.ab,kf,ti. \| 20674 \| \| 3 \| 1 or 2 \| 25386 \| \| 4 \| ((core adj3 (outcome* or set or sets)) or "outcome report*" or "outcome term*").ab,kf,ti. \| 8248 \| \| 5 \| 3 and 4 \| 10 \| \| **6** \| **limit 5 to (yr="2000 -Current" and english)** \| **9** \|   *ab=abstract, kf=author keyword, ti=title*  **Database:** The Cochrane Library **Date:** 2021-02-10 **No of results:** 1 ref   \| **ID** \| **Search** \| **Hits** \| \| --- \| --- \| --- \| \| #1 \| MeSH descriptor: [Appendicitis] explode all trees \| 589 \| \| #2 \| (appendicitis):ti,ab,kw (Word variations have been searched) \| 1589 \| \| #3 \| #1 OR #2 \| 1589 \| \| #4 \| ((core NEAR/3 (outcome* OR set OR sets)) OR ”outcome report*” OR ”outcome term*”):ti,ab,kw (Word variations have been searched) \| 1588 \| \| #5 \| #3 AND #4 \| 2 \| \| **#6** \| **Limit search to publication year 2000-2021** \| **1** \|   *ti=title, ab=abstract, kw=keywords*  **Database:** Embase (Ovid) 1974 to 2021 February 09 **Date:** 2021-02-10 **No of results:** 14 ref   \| **#** \| **Searches** \| **Results** \| \| --- \| --- \| --- \| \| 1 \| exp appendicitis/ \| 25352 \| \| 2 \| appendicitis.ab,kw,ti. \| 21707 \| \| 3 \| 1 or 2 \| 28885 \| \| 4 \| ((core adj3 (outcome* or set or sets)) or "outcome report*" or "outcome term*").ab,kw,ti. \| 11373 \| \| 5 \| 3 and 4 \| 15 \| \| **6** \| **limit 5 to (english and yr="2000 -Current")** \| **14** \|   *ab=abstract, kf=author keyword, ti=title*  **Database:** Cinahl (EBSCO) **Date:** 2021-02-10 **No of results:** 3 ref   \| **#** \| **Undran** \| **Resultat** \| \| --- \| --- \| --- \| \| **S6** \| **S3 AND S4**  **Avgränsare - Publiceringsdatum: 20000101-20210231; Språk: English Utökning - Sök med relaterade ord; Sök med likvärdiga ämnesord** \| **3** \| \| S5 \| S3 AND S4 \| 3 \| \| S4 \| TI ( (core N3 (outcome* OR set OR sets)) OR "outcome report*" OR "outcome term*" ) OR AB ( (core N3 (outcome* OR set OR sets)) OR "outcome report*" OR "outcome term*" ) OR SU ( (core N3 (outcome* OR set OR sets)) OR "outcome report*" OR "outcome term*" ) \| 3,474 \| \| S3 \| S1 OR S2 \| 4,672 \| \| S2 \| TI appendicitis OR AB appendicitis OR SU appendicitis \| 4,672 \| \| S1 \| (MH "Appendicitis") \| 3,533 \|   *TI=title, AB=abstract, SU=Keywords, Major Subjects and Minor Subjects, MH=Exact Subject Headings* | | |  |
| --- | --- | --- | --- | --- | --- | --- | --- | --- | --- | --- | --- | --- | --- | --- | --- | --- | --- | --- | --- | --- | --- | --- | --- | --- | --- | --- | --- | --- | --- | --- | --- | --- | --- | --- | --- | --- | --- | --- | --- | --- | --- | --- | --- | --- | --- | --- | --- | --- | --- | --- | --- | --- | --- | --- | --- | --- | --- | --- | --- | --- | --- | --- | --- | --- | --- | --- | --- | --- | --- | --- | --- | --- | --- | --- | --- | --- | --- | --- | --- | --- | --- | --- | --- | --- | --- | --- | --- |
| Supplement References 1. Cogley, J. R., et al. (2012). "Emergent pediatric US: what every radiologist should know." Radiographics 32(3): 651-665.    2. Dahabreh, I. J., et al. (2015). Agency for Healthcare Research and Quality 15(16): 12.    3. Fedko, M., et al. (2014). "Ultrasound evaluation of appendicitis: importance of the 3 x 2 table for outcome reporting." American Journal of Emergency Medicine 32(4): 346-348.    4. Hall, N. J., et al. (2015). "Outcome reporting in randomised controlled trials and meta-analyses of appendicitis treatments in children: a systematic review." Trials [Electronic Resource] 16: 275.    5. Hall, N. J., et al. (2017). "Incorporating the views of children and parents into a core outcome set for the treatment of acute appendicitis in children." Journal of Evidence-Based Medicine 10 (Supplement 1): 36.  6. Knaapen, M., et al. (2019). "Protocol for the development of a global core outcome set for reporting treatment of uncomplicated appendicitis in children." Journal of Evidence-Based Medicine 12 (Supplement 1): 15.  7. Knaapen, M., et al. (2019). "Establishing a core outcome set for treatment of uncomplicated appendicitis in children: study protocol for an international Delphi survey." BMJ Open 9(5): e028861.  8. Mason, A., et al. (2012). "How well do diagnosis-related groups for appendectomy explain variations in resource use? An analysis of patient-level data from 10 European countries." Health Economics (United Kingdom) 21(SUPPL. 2): 30-40.  9. Mukhopadhyay, S., et al. (2017). "Lancet commission on global surgery." Iranian Journal of Pediatrics 27 (4) (no pagination)(e11273).  10. Ruperto, N., et al. (2016). "Prednisone versus prednisone plus ciclosporin versus prednisone plus methotrexate in new-onset juvenile dermatomyositis: A randomised trial." The Lancet 387(10019): 671-678.  11. Sherratt, F. C., et al. (2020). "Core outcome set for uncomplicated acute appendicitis in children and young people." British Journal of Surgery 107(8): 1013-1022.  12. Sherratt, F. C., et al. (2019). "Improving core outcome set development for children and young people: Learning from a case study in acute appendicitis and consultation with an international group of children and young people." Journal of Evidence-Based Medicine 12 (Supplement 1): 21.  13. Sherratt, F. C., et al. (2019). "Development of a core outcome set for acute uncomplicated appendicitis in children and young people." Journal of Evidence-Based Medicine 12 (Supplement 1): 12-13.  14. Sherratt, F. C., et al. (2017). "Development of a core outcome set to determine the overall treatment success of acute uncomplicated appendicitis in children: a study protocol." BMJ Paediatrics Open 1(1): e000151.  15. Yousef, Y., et al. (2018). "Risk stratification in pediatric perforated appendicitis: Prospective correlation with outcomes and resource utilization." Journal of Pediatric Surgery 53(2): 250-255. |  |  |  |
